# MicroRNA-29b Plays a Vital Role in Podocyte Injury and Glomerular Diseases through Inducing Mitochondrial Dysfunction

**DOI:** 10.7150/ijbs.93506

**Published:** 2024-09-03

**Authors:** Jiafeng Liu, Yabing Xiong, Hongyan Mo, Hongxin Niu, Jinhua Miao, Weiwei Shen, Shan Zhou, Xiaoxu Wang, Xiaolong Li, Yunfang Zhang, Kunling Ma, Lili Zhou

**Affiliations:** 1 State Key Laboratory of Organ Failure Research, National Clinical Research Center of Kidney Disease, Guangdong Provincial Clinical Research Center for Kidney Disease, Guangdong Provincial Key Laboratory of Nephrology, Division of Nephrology, Nanfang Hospital, Southern Medical University, Guangzhou, China.; 2 Department of Nephrology, the Second Affiliated Hospital, School of Medicine, Zhejiang University, Hangzhou, China.; 3 Department of Nephrology, the First Affiliated Hospital, Hengyang Medical School, University of South China, Hengyang, Hunan, China.; 4 Special Medical Service Center, Zhujiang Hospital, Southern Medical University, Guangzhou, China.; 5 Department of Nephrology, Huadu District People's Hospital, Southern Medical University, Guangzhou, China.

**Keywords:** diabetic kidney disease (DKD), podocyte, mitochondria, PGC-1α, miR-29b

## Abstract

Diabetic kidney disease (DKD) is becoming the most leading cause of end-stage renal disease (ESRD). Podocyte injury plays a critical role in DKD progression. Notably, mitochondrial dysfunction is crucial for podocyte injury. MicroRNAs (miRNAs) involves in various kidney diseases. Herein, we discovered miR-29b was induced in the urine of 126 patients with DKD (stage I and II), and negatively correlated with kidney function and podocyte homeostasis. Mechanically, miR-29b targeted peroxisome proliferator-activated receptor-γ coactivator-1α (PGC-1α), a co-activator of transcription factors regulating mitochondrial biogenesis and energy metabolism. In vitro, ectopic miR-29b downregulated PGC-1α and promoted podocyte injury, while inhibition of miR-29b alleviated podocyte injury. Consistently, inhibition of miR-29b mitigated podocyte injury and preserved kidney function in ADR nephropathy and db/db mice, and overexpression of miR-29b accelerated disease. Knockout miR-29b specifically in podocyte inhibited mitochondrial dysfunction and podocyte injury. These results revealed miR-29b plays a crucial role in mitochondrial dysfunction through targeted inhibition on PGC-1α, leading to podocyte injury and DKD progression. Importantly, miR-29b could serve as a novel biomarker of podocyte injury and assists to early diagnose DKD.

## Introduction

Diabetic kidney disease (DKD) is the common complication of diabetic mellitus, also the most leading cause of end-stage renal disease (ESRD) worldwide^1^, ^2^. Except glucose and blood pressure control, the therapeutic strategies of DKD are quite limited^3^. Growing evidence shows podocyte injury plays a critical role in DKD^4^-^6^. As highly specialized, terminally differentiated cells, podocytes are responsible to molecular and charge barriers to accomplish glomerular filtration function^7^. Of note, once injured, podocyte's homeostasis is very hard to be restored. Hence, it is of great value to identify the early biomarkers of podocyte injury and explore the effective intervention strategies.

Mitochondrion is a vital organelle for maintaining cellular metabolism and energy homeostasis^8^. Podocytes are also rich with mitochondria and strongly depend on them to maintain cellular stability. Large amounts of studies demonstrate mitochondrial dysfunction is a crucial factor for podocyte injury, especially in DKD^9^-^12^.

PGC-1α, a coactivator of transcription factors, is a key player in regulating mitochondrial biogenesis and energy metabolism. PGC-1α binds to transcription factors NRF1 and NRF2, and then triggers the transcription of nuclear gene encoding mitochondrial transcription factor A (TFAM), an important transcription factor regulating mitochondrial biogenesis. PGC-1α can also bind with PPARα, a nuclear gene encoding fatty acid β-oxidation (FAO)-related genes, such as CPT1a, CPT2 and Acox1, etc. In addition, PGC-1α also regulates mitochondrial antioxidant enzymes such as MnSOD, catalase, peroxide reductase 3 (Prx3) and Prx5, and also involves in energy metabolism processes such as glucose uptake, gluconeogenesis, and adipogenesis^13^-^16^. Studies have shown overexpression of PGC-1α ameliorates podocyte injury via enhancing mitochondria mass, cellular respiration and ATP production, indicating PGC-1α plays a protective role in podocytes through preserving mitochondrial function^12^, ^17^, ^18^.

MicroRNAs (miRNAs) are endogenous, non-coding single-stranded small RNAs with a length of about 22 nucleotides (nt). miRNAs are widely present in eukaryotes. Through binding to the 3'-untranslated region (3'-UTR) of targeted mRNA, miRNAs negatively regulate targeted gene at post-transcriptional level 19, 20. MicroRNAs highly involve in various organ damages, as well as kidney diseases^21^-^23^. However, their roles in podocyte injury and the underlying mechanisms have not been elucidated in detail.

MicroRNA-29 family is highly conserved in mammals' evolution, comprised with 3 mature members, including miR-29a, b and c, which are generated from two primary transcripts: pri-miR-29a/b1 cluster and pri-mir-29b2/c cluster. As reported, the miR-29 family acts as key regulators in both biological processes and disease. Reports show miR-29b involves in tumor, osteoarthritis, osteoporosis, and immune disease^19^, and also, plays a role in heart, lung, liver, as well as kidney diseases 24-26. However, we found miR-29 was greatly upregulated at the early stage of podocyte injury, which was inconsistent with its downregulation in fibrotic organs including kidney. These prompted us to further explore the role of miR-29 in podocyte injury.

In this study, we found that miR-29b was upregulated in glomeruli in the early stage of DKD. MiR-29b specifically targeted PGC-1α mRNA and inhibited its expression, leading to mitochondrial dysfunction and podocyte injury. Urinary miR-29b also served as a novel biomarker of podocyte injury and early diagnostic biomarker of DKD. Targeted inhibition of miR-29b was also a new and promising therapeutic strategy for the prevention of DKD and glomerular diseases.

## Methods

### Human urine samples and kidney biopsies

Human urine samples and the kidney biopsies were collected from patients with newly diagnosis of DKD. The demographic and clinical data are presented in Supplementary Table S1. All the studies involving human samples were performed with informed patient consent and approved by the Medical Ethics Committee of Huadu District People's Hospital, Southern Medical University (ethic approval no. 2023115).

### miRNA in situ hybridization

In situ hybridization for miR-29b transcripts was performed using Digoxigenin-labeled LNA-miR-29b probes (Exiqon, Vedbaek, Denmark) and Enhanced Sensitive ISH Detection kit II (AP) (Boster, Pleasanton, CA) according to the manufacturer's protocol.

### Urinary albumin, nephrin, N-gal and creatinine assay

Urinary albumin was determined by a mouse albumin enzyme-linked immunosorbent assay quantitation kit (CSB-E13878m; CUSABIO). Urinary creatinine (DICT-500; BioAssay Systems) was measured followed by a routine procedure. Urinary albumin was normalized to creatinine and presented as mg albumin/mg creatinine. Urinary nephrin (1035; Ethos Biosciences) and N-gal (SEB388Hu; Ethos Biosciences) were measured according to the manufacturer's protocol by enzyme-linked immunosorbent assay quantitation kit.

### Isolation and quantification of mtDNA

Human urine supernatants were concentrated using Amicon Ultracel-30k centrifugal filters (EMD Millipore, San Diego, CA). DNA was isolated from the concentrate by a Viral RNA Mini Kit (Qiagen, Valencia, CA) and was determined using the Quant-iT PicoGreen dsDNA reagent (Life Technologies, Carlsbad, CA). qPCR was used for the mitochondrial gene ND1 and the nuclear gene β-actin to determine UmtDNA content with a template input of 0.3-5 ng of total DNA. mtDNA levels in urine were corrected by urine creatinine. As for kidney tissues and cells, DNA was extracted by DNeasy Blood & Tissue Kit (GIAGEN). qPCR was used for the mitochondrial gene COX2 and RSP18 to determine mtDNA content with a template input of 0.3-5 ng of total DNA.

### Quantitative real-time PCR

Total RNA was extracted with TRIzol reagent (Invitrogen, Carlsbad, CA). The RNA was reverse transcribed using the Reverse Transcription System Kit (R323-01; Vazyme) according to the instructions of the manufacturer. Real-time PCR was performed using a qPCR SuperMix kit (Q341-02; Vazyme).

For determining the levels of miRNAs, RNA was extracted from glomeruli and human urine by miRNeasy Serum/Plasma Kit (GIAGEN, Wuhan, China). And then RNA was reversely transcribed by TaqMan microRNA Reverse Transcription Kit (4366596; Applied Biosystems). TaqMan microRNA Assay for hsa/mmu-miR-29a/b/c was performed according to the instructions of manufacturers (4440047; Applied Biosystems). U6 RNA was used for normalization.

### Reporter vector and luciferase assays

The 3′-UTR of PGC-1α was obtained from mouse genomic DNA by PCR and cloned into the psiCHECK-2 vector (Promega, Madison, WI). Mutated PGC-1α 3′UTR vectors containing mutations corresponding to miR-29b seed sequence were also constructed, from TCGTGGT to CTACAAC. The constructs, psiCHECK-2- PPARGC1A -WT and psiCHECK-2 PPARGC1A -Mutant were sequence verified and co-transfected with control or miR-29b mimics into 293T cells by lipofectamine 2000. Luciferase assay was performed using a dual luciferase assay system kit according to the manufacturer's protocols (Promega, Madison, WI).

### Western blot

Protein expression was assessed by Western blot analyses as described previously. Primary antibodies used in experiments were as follows: ZO-1 (QF215185; Life Technologies, Carlsbad, CA), podocalyxin (AF1556; R&D Systems), Desmin (PB0095; Boster, Wuhan, China), nephrin (ab58968; Abcam, Cambridge, UK), WT1 (sc-393498; Santa Cruz Biotechnology, Dallas, TX), PGC-1α (66369; proteintech), TFAM (GTX112760; Genetex), COX1 (SAB1301619; Sigma-Aldrich), TOMM20 (ab186735; Abcam, Cambridge, UK), Cytb (SAB1304939; Sigma-Aldrich), fibronectin (F3648; Sigma-Aldrich), anti-GAPDH (RM2001; Ray Antibody Biotech) and α-tubulin (BM3885; Boster, Wuhan, China).

### Immunofluorescence staining

Kidney cryosections (3μm) or cells cultured on coverslips were fixed with 4% paraformaldehyde for 15 min at room temperature. After blocking with 10% donkey serum for 60 min, the slides were immunostained with primary antibodies against fibronectin (F3648; Sigma-Aldrich), WT1 (sc-393498; Santa Cruz Biotechnology, Dallas, TX), ZO-1 (QF215185; Life Technologies, Carlsbad, CA), podocalyxin (AF1556; R&D Systems), nephrin (ab58968; Abcam, Cambridge, UK), Synaptopodin (sc515842; Santa Cruz Biotechnology, Dallas, TX), TOMM20 (ab186735; Abcam, Cambridge, UK), ADRP (ab108323; Abcam, Cambridge, UK), PGC-1α (66369-1; proteintech), Flag (M185-3; MBL). The slides were then incubated with secondary antibodies (Jackson Immuno-Research Laboratories) and augmented by Vectashield antifade mounting media (Vectorlab).

### Measurements of mitochondrial oxygen consumption rate

The rates of mitochondrial oxygen consumption in MPC5 cells administrated by miR-29b mimic or high glucose combined with miR-29b inhibitor were measured with a Seahorse XFe96 Extracellular Flux Analyzer (Seahorse Bioscience). OCR was assessed using the XF Cell Mito Stress Test Kit (#103015; Seahorse Bioscience) according to the manufacturer's protocol. In brief, approximately 3 × 10^4^ cells were seeded per well in a Seahorse 96-well XF Cell Culture Microplate. XF assays were performed with the addition of oligomycin (1μmol/L), FCCP (0.5μmol/L) and antimycin A and rotenone (0.5μmol/L). Data were analyzed on Agilent Seahorse Analytics, a software platform. Seahorse Analytics automatically calculates oxidative stress test parameters: basal respiration, acute response to inhibitors, maximal respiration and so on.

### Animal models

Male BALB/c mice at 6 weeks of age were purchased from the Animal Center of Southern Medical University (Guangzhou, China). Mouse model of podocyte injury and proteinuria was established by an intravenous injection of ADR (11.5 mg/kg) (doxorubicin hydrochloride; Sigma-Aldrich). Saline injection was applied to control group. BALB/c mice were randomly divided into three different groups: (i) control mice injected with saline; (ii) ADR mice injected with antagomir NC (350μg); (iii) ADR mice injected with miR-29b antagomir (350μg).

For DKD model, male obesity and type 2 diabetic db/db mice and their lean and nondiabetic db/m controls were raised up to the age of 12 weeks or 20 weeks. To knockdown miR-29b expression, we injected the miR-29b antagomiR (GenePharma, Shanghai, China) or control antagomiR into mice via the tail vein by injection of 50 μg per mice each time.

For overexpression of miR-29b mice model, BALB/c mice were randomly divided into three different groups: (i) control mice injected with pcDNA3 vector; (ii) mice injected with pCMV-pri-mir-29b (1mg/kg); (iii) ADR mice injected with pCMV-pri-mir-29b (1mg/kg) and oral administration of Resveratrol (400mg/kg). The pCMV-pri-mir-29b plasmid was injected intravenously by a hydrodynamic-based gene delivery approach as previously reported. All animal experiments were approved by the Animal Ethics Committee of Nanfang Hospital, Southern Medical University.

### Generation of miR-29b^flox/flox^ mice

The miR-29b^flox/flox^ mice were generated in C57BL/6 background by CRISPR/ Cas9 system and produced in Cyagen (Cyagen Biosciences Inc, China).

### Isolation of glomeruli

Glomeruli from db/db mice and ADR mice were isolated according to the manufacturer's protocol. Briefly, mice were sacrificed and perfused with Dynabead M-450 (00388551, Invitrogen). The kidneys were minced and digested in collagenase IV (1 mg/ml, Invitrogen), and pressed through a 100-μm cell strainer (BD Falcon, Bedford, MA), and then glomeruli were gathered using a magnetic concentrator.

For isolation from miR-29b^flox/flox^ mice, mice were sacrificed in a sterile environment. Kidneys were carefully harvested, decapsulated, minced, and then smashed down carefully through three sieves sequentially: 100-μm, 70-μm, 40-μm cell strainer (BD Falcon, Bedford, MA). After PBS washing and centrifugation, the glomeruli of miR-29b^flox/flox^ mice were resuspended in RPMI1640 with 10% FBS medium and plated on noncoated six-well plates for next step.

### Cell culture and treatment

Mouse podocyte cell line (MPC5) and human embryonic kidney cells (293T) were cultured as described. MPC5 were transfected with miRNA mimics or miRNA inhibitor or β-catenin expression plasmid (pDel-β-catenin). Opti-MEM medium and Lipofectamine 2000 (Invitrogen, Carlsbad, CA) were mixed according to the manufacturer's instructions. MPC5 cells were treated with high glucose (25mmol/L) for 48 hours.

Mini-organ glomeruli from miR-29b^flox/flox^ mice were resuspended in RPMI1640 with 10% FBS and transfected with adenovirus expressing NPHS2-cre recombinase (AdV-NPHS2-Cre) (Cyagen Biosciences) to knockout miR-29b specially in podocytes. Glomeruli were treated with adenovirus for 48 hours and followed by high glucose (25mmol/L) treatment for another 48 hours.

### MitoSOX, and MitoTracker staining

Frozen sections (3μm) and cultured cells on coverslips were used for detection of mitochondria mass via MitoTracker deep red (M22426; Thermo Fisher) or mitochondrial ROS production via mitoSOX (M36008; Thermo Fisher) staining according to the manufacturer's instructions.

### Flow cytometry analysis

MPC5 cells were seeded on 6-well plates and were given the high glucose treatment before staining. Then, cells were trypsinized and suspended with 400μl of JC-1s solution (5μmol/L, T3168; Thermo Fisher) and were incubated at 37°C in the dark for at least 30 min. Then, cells were centrifuged, washed once, and resuspended in 400μl of PBS. The cells were analyzed in a flow cytometry analyzer (BD FACS Calibur System). The relative MMP was measured by the ratio of J-aggregate/ monomer (590/520 nm).

### Histology and immunohistochemical staining

Kidney tissue sections were subjected to periodic acid-Schiff (PAS) using standard protocols. Immunohistochemical staining was performed as described previously. After incubation with specific antibodies, sections were stained using Vector M.O.M. Immunodetection Kit according to the manufacture's protocol (Vector Laboratories). Antibodies used were as follows: fibronectin (F3648; Sigma-Aldrich), PGC-1α (66369-1; proteintech).

### F-actin staining

Cultured cells on coverslips were incubated with a F-actin (72485; Sigma-Aldrich) dye for 40 min and DAPI (Sigma-Aldrich) for 10 min. Images were taken by a Leica TCS-SP8 confocal microscope.

### Transmission electron microscopy

For electron microscopy to assess podocyte injury, renal cortex tissue blocks (about 1mm^3^) were carefully cut and placed in fixed solution. The ultrastructure of podocytes and mitochondria were observed by transmission electron microscope (JEM-400 Plus, JEOL, Tokyo, Japan).

### Statistical analyses

All data were presented as means ± SEM. Statistical analyses of the data were performed using SPSS 20.0 (SPSS Inc., Chicago, IL). Differences between groups was made using t test or one-way ANOVA followed by Dunnett's T3. P < .05 was considered statistically significant.

## Results

### MiR-29b is induced in podocytes and activated in the early stage of glomerular injury

**Figure 1:**
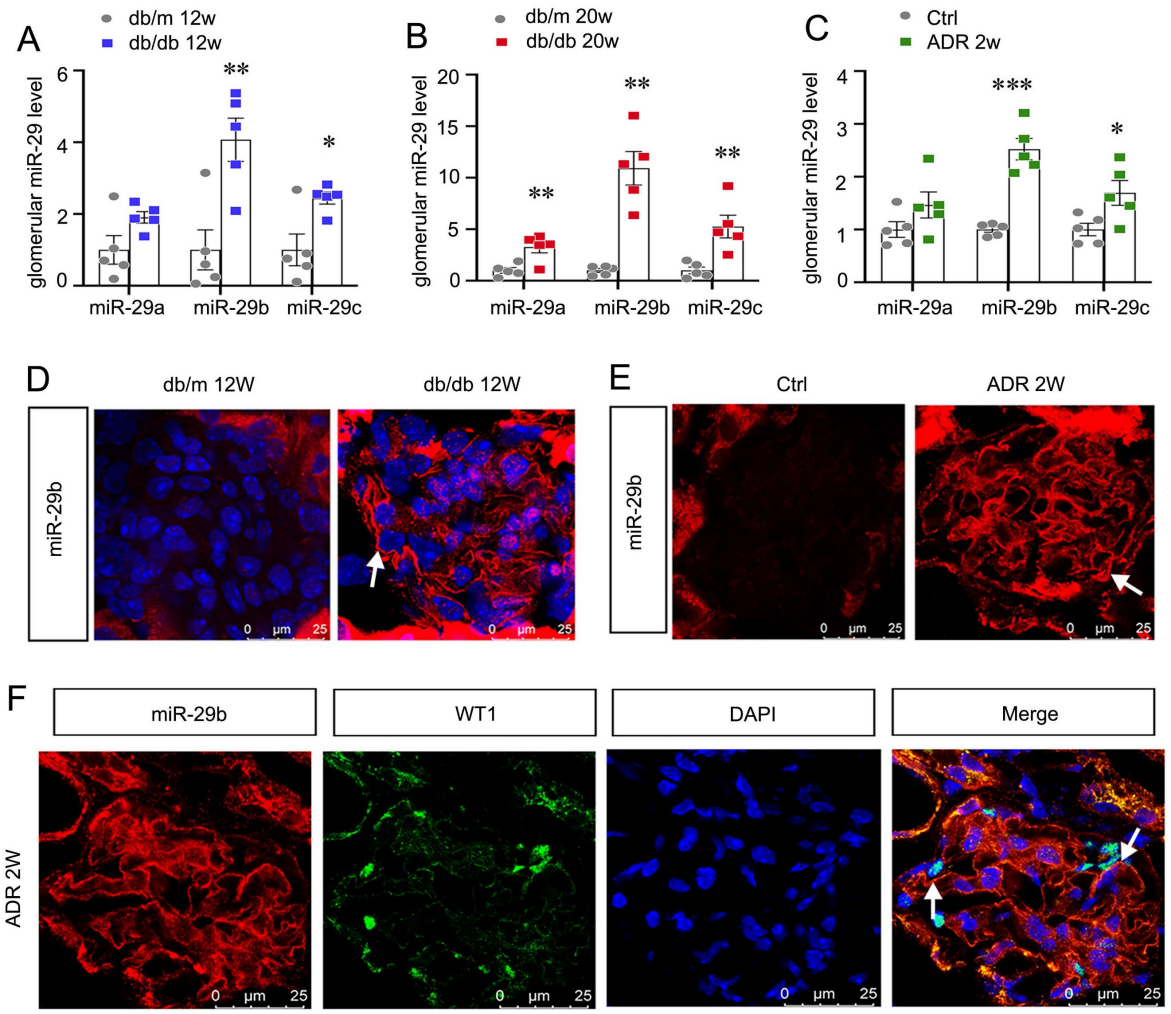
** MiR-29b is induced in podocytes and activated in the early stage of glomerular injury.** (A-C) Graphic presentation shows the expression changes of the miR-29a/b/c in glomeruli of db/db mice and ADR mice. *P < 0.05, **P < 0.01, ***P < 0.001 versus control group (n=5). (D-E) In situ hybridization shows the localization of miR-29b in glomerular podocytes in db/db mice at 12 weeks and mice at 2 weeks after injection of ADR. Arrows indicate positive staining. Bar = 25μm. (F) Co-staining of miR-29b and WT1 in glomerular podocytes in mice at 2 weeks after injection of ADR. Arrows indicate positive miR-29b staining in podocytes. Bar = 25μm.

We first assessed miR-29s levels in db/db mice, the type 2 diabetes model. As show in Figure 1A and B, among miR-29s family, miR-29b was the most significantly increased miRNA in isolated glomeruli in db/db mice at 12 or 20 weeks of age, an early stage of DKD. We also assessed the expression of miR-29s family in isolated glomeruli from adriamycin nephropathy (ADR) mice. As shown in Figure 1C, miR-29b was also increased most among miR-29s family in mice with ADR injection for 2 weeks, a glomerular disease model with severe podocyte injury. MiR-29b was then examined by in situ hybridization (ISH). Compared with the negative signal in db/m or control mice, a strong staining of miR-29b was discovered in glomeruli in db/db and ADR mice (Figure 1D and E). We then performed the co-staining of miR-29b with WT1, a specific marker of podocyte. As shown, the expression of miR-29b was highly co-localized with WT1, suggesting its location in podocytes (Figure 1F).

### MiR-29b level is associated with loss of kidney function and podocyte injury in human DKD

**Figure 2:**
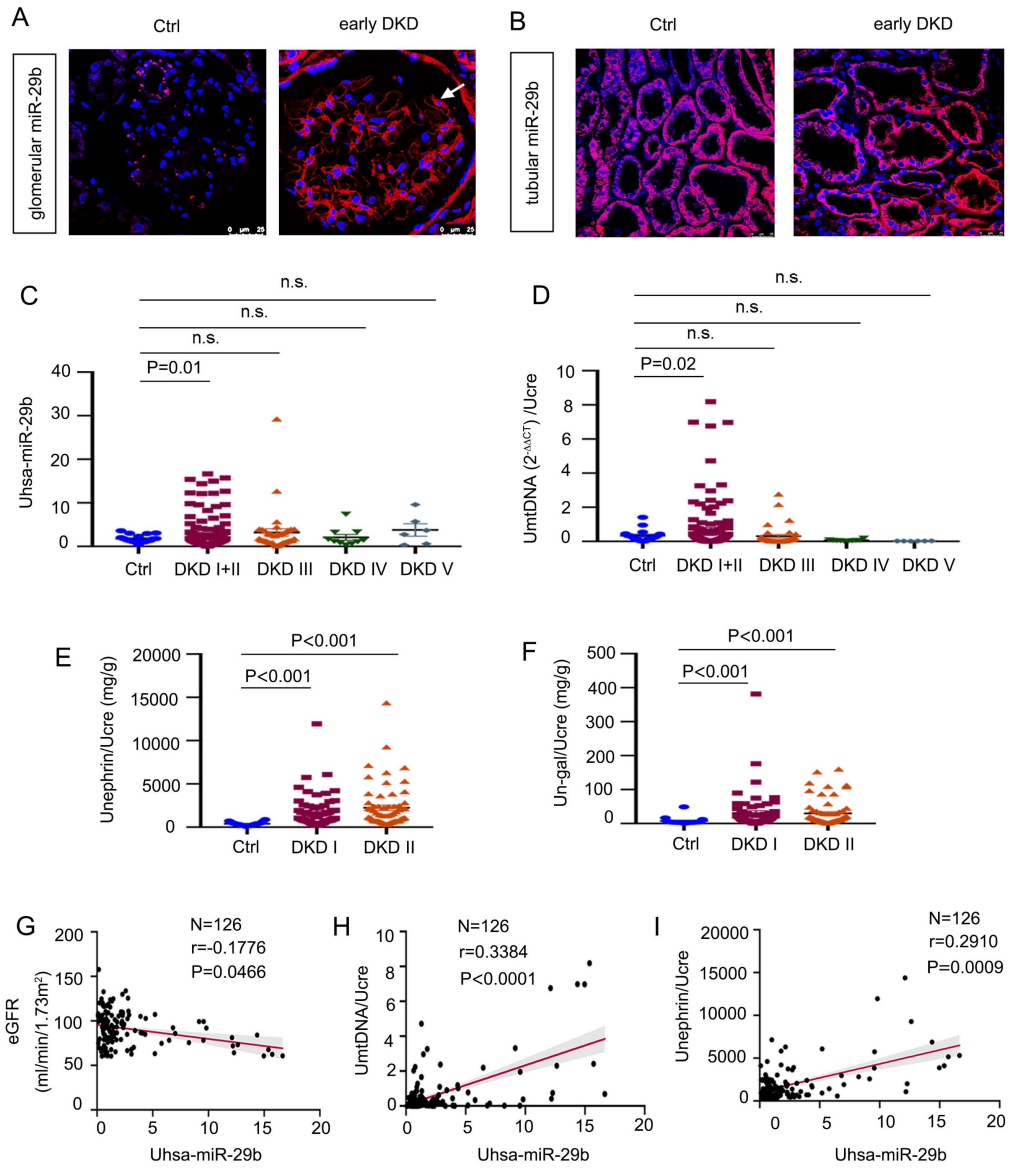
** MiR-29b level is associated with loss of kidney function and podocyte injury in human DKD.** (A-B) In situ hybridization shows the localization of miR-29b in glomerular podocytes and tubular cells in DKD I-II stage patients. Arrows indicate positive miR-29b staining in podocytes. Bar = 25μm. (C) Graphic presentation shows urinary miR-29b level in a cohort of patients with DKD (n = 178) and healthy participants (n = 18). (D-F) Graphic presentation shows urinary mtDNA, nephrin, and N-gal in different stages of DKD (n = 178, DKD I-V stage; n = 126, DKD I-II stage) and healthy participants (n = 18). Data was normalized to urinary creatinine and presented as l/g creatinine and mg/g creatinine. (G) Linear regression shows a negative correlation between urinary miR-29b and estimated glomerular filtration rate (eGFR) (n = 126, DKD I-II stage). (H-I) Linear regression shows a positive correlation between urinary miR-29b and mtDNA, miR-29b and nephrin level in urine (n = 126, DKD I-II stage).

To explore the relevance of miR-29b in human DKD, we first examined the expression of miR-29b in kidney biopsies from patients with early DKD by ISH. As shown in Figure 2A-B, miR-29b was greatly increased in podocytes, but there was no change in tubules. We then measured urinary miR-29b level in 18 healthy subjects and 178 patients with DKD. The detailed demographic and clinical data of the patients are presented in Supplementary Materials. As show in Figure 2C, miR-29b level in urine was elevated in patients with DKD, especially in the early stages of disease, such as DKD stage 1 and 2. Besides, mtDNA in urine was also significantly increased in patients with early stage of DKD (stage 1 and 2) (Figure 2D). We further assessed the urinary level of nephrin, a well-known podocyte marker, and the urinary level of N-gal, a tubular cell injury marker. A shown in Figure 2, E and F, both urinary nephrin and N-gal were significantly increased in the early stages of DKD. We next analyzed the correlation between urinary levels of miR-29b and estimated glomerular filtration rate (eGFR), a marker for kidney function. As show in Figure 2G, urinary miR-29b was inversely correlated with eGFR. We then assess the correlation of urinary levels of miR-29b with urinary mtDNA and nephrin. As shown in Figure 2H and I, urinary expression of miR-29b was positively correlated with urinary levels of mtDNA and nephrin in DKD patients. We also examined the correlation between urinary miR-29b and urinary N-gal and found that they were not correlated significantly (Figure S2 L).

### MiR-29b promotes podocyte injury by targeting PGC-1α

**Figure 3:**
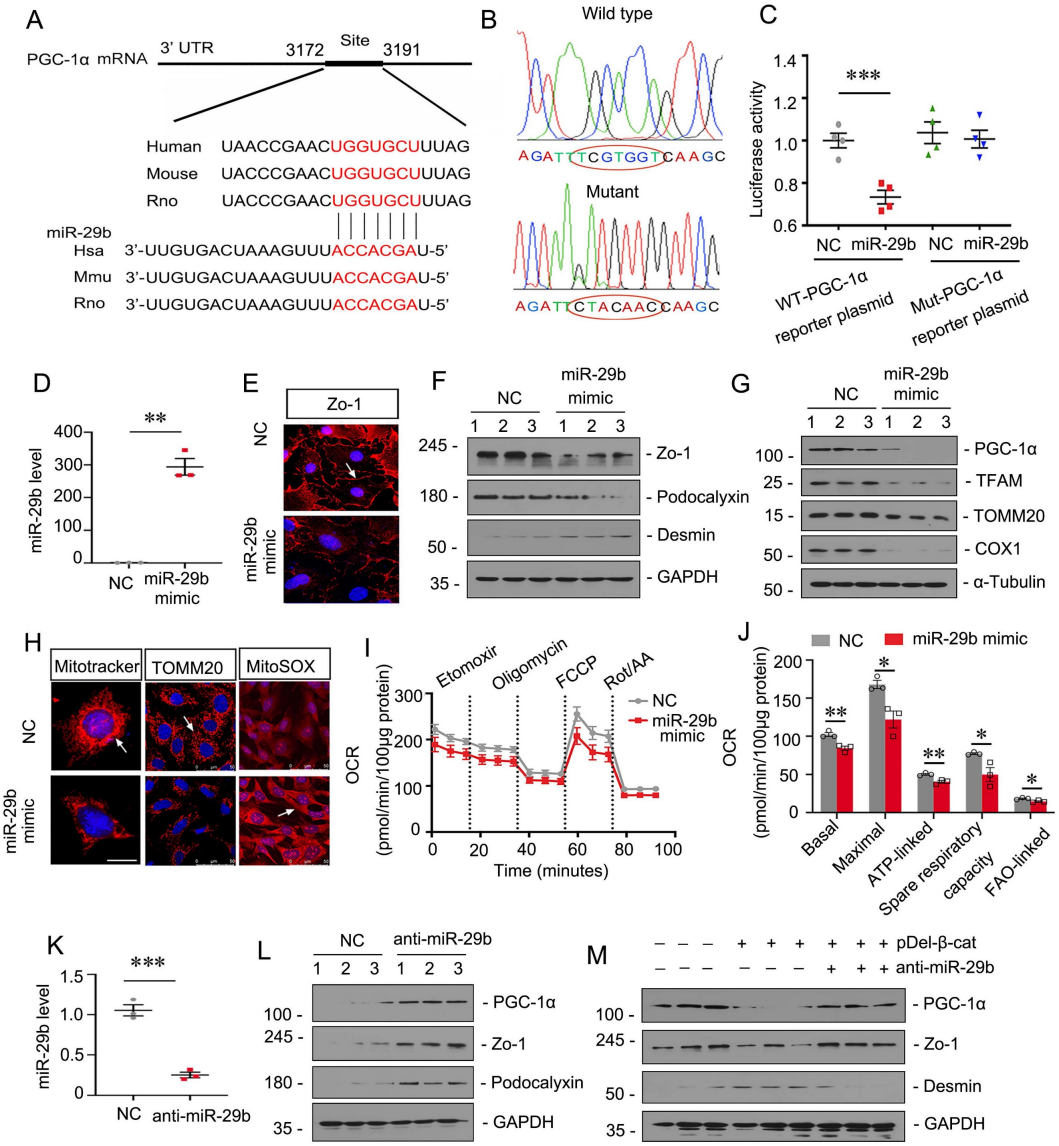
** MiR-29b promotes podocyte injury by targeting PGC-1α.** (A) Bioinformatics analysis shows the predicted binding sites of miR-29b in the PGC-1α 3′-untranslated region (UTR) using the TargetScan software. (B) Sequence validation of the wild type or mutant PGC-1α 3′-UTR for the luciferase reporter construction. The wild-type miR-29b binding site in PGC-1α 3′-UTR (upper) and the mutated one (bottom) in the region corresponding to the miR-29b seed sequence are shown. (C) Luciferase reporter assay show that miR-29b decreased the luciferase activity in 293T cells co-transfected with wild-type PGC-1α 3′ UTR, but not with mutant PGC-1α 3′ UTR. ***P < 0.001 versus control group (n=4). (D) Mouse podocytes (MPC5) were transfected with miR-29b mimic or negative control (miR-Ctrl, NC) for 24h. qRT-PCR analysis shows the relative levels of miR-29b. **P < 0.01 versus control group (n=3). (E) Immunostaining of ZO-1 were presented. Arrows indicate positive staining. Bar = 25μm. (F) Representative western blot showing expression of ZO-1, podocalyxin and Desmin in two groups. Numbers (1-3) indicate each individual culture in each given group. (G) Representative western blot showing expression of PGC-1α, TFAM, TOMM20 and COX1 in two groups. Numbers (1-3) indicate each individual culture in each given group. (H) Representative micrographs show mitotracker staining, immunostaining of TOMM20 and mitoSOX probe staining. Arrows indicate positive staining. Bar = 20μm or 50μm. (I-J) Graphical representations of basal OCR, maximal OCR, ATP-linked OCR, spare respiratory capacity and FAO-linked OCR in different groups. *P < 0.05, **P < 0.01 versus control group (n=3). (K) MPC5 cells were transfected with miR-29b inhibitor (anti-miR-29b) or control (anti-miR-Ctrl, NC) for 24 h. qRT-PCR analysis shows the relative levels of miR-29b.***P < 0.01 versus control group (n=3). (L) Representative western blot showing expression of PGC-1α, Zo-1 and podocalyxin in two groups. Numbers (1-3) indicate each individual culture in each given group. (M) MPC5 cells were transfected with pDel-β-catenin or co-transfected with miR-29b inhibitor for 24 h. Representative western blot showing expression of PGC-1α, Zo-1 and Desmin among three groups. Numbers (1-3) indicate each individual culture in each given group.

Through bioinformatics analyses from TargetScan, we found miR-29b could target PGC-1α by binding to the 3′-untranslated regions (UTR) of PGC-1α mRNA, which harbored the conserved complementary sites to the seed sequence of miR-29b (Figure 3A). To determine whether PGC-1α is a direct target of miR-29b, we constructed the luciferase reporter plasmids containing 3′-UTR (wild type) or mutant sequence of PGC-1α corresponding to the seed sequence of miR-29b (Figure 3B). These plasmids were transfected into 293T cells in combination with miR-29b mimic or control miRNA (NC). As shown in Figure 3C, transfection with miR-29b inhibited the luciferase reporter activity in wild-type PGC-1α, plasmid but not in PGC-1α plasmid with the mutant 3′- UTR, suggesting that PGC-1α is a direct target of miR-29b.

To study the potential role of miR-29b in podocyte injury, we transfected miR-29b mimic or inhibitor into mouse podocyte cell line (MPC5). As shown in Figure 3D, a marked increase in miR-29b expression in MPC5 cells was confirmed by TaqMan probe-based qPCR. Transfection with miR-29b mimic markedly decreased the expression of Zo-1, a marker of podocyte integrity (Figure 3E). Similar results were observed when the protein levels of Zo-1 and podocalyxin were assessed by Western blot (Figure 3F). Moreover, the expression of Desmin, a marker of podocyte injury, was induced by overexpression of miR-29b (Figure 3F). We then assessed mitochondria-related proteins. As shown in Figure 3G, transfection of miR-29b substantially inhibited PGC-1α expression in MPC5 cells, as well as TFAM, a key transcription factor for mitochondrial biogenesis. The expression of mtDNA-encoded oxidative phosphorylation (OXPHOS) complex IV subunit cytochrome c oxidase 1 (COX1) and the outer mitochondrial membrane protein translocase (TOMM20) was also examined. As shown in Figure 3G, overexpression of miR-29b greatly decreased their expression. We then assessed mitochondrial mass by Mitotracker labeling fluorescence and immunostaining for TOMM20 protein. Results showed that overexpression of miR-29b induced mitochondrial mass loss (Figure 3H). We then performed mitoSOX probe staining. The results showed miR-29b greatly induced mitochondrial ROS production (Figure 3H). To further investigate mitochondrial function, we performed oxygen consumption rate (OCR) analysis by Seahorse assay. As shown in Figure 3I and J, miR-29b significantly reduced the levels of basal OCR, maximal OCR, ATP-linked OCR, spare respiratory capacity and FAO-linked OCR.

In contrast, inhibition to miR-29b in MPC5 cells upregulated the expression of PGC-1α, Zo-1 and podocalyxin (Figure 3K and L). Moreover, inhibition to miR-29b also reversed the expression of PGC-1α and Zo-1, but decreased Desmin, although they were induced by pDel-β-catenin transfection in MPC5 cells (Figure 3M). These results suggest that miR-29b targets PGC-1α to further induce mitochondrial dysfunction and promote podocyte injury.

### Inhibition of miR-29b mitigates high glucose-induced podocyte injury by increasing PGC-1α

**Figure 4:**
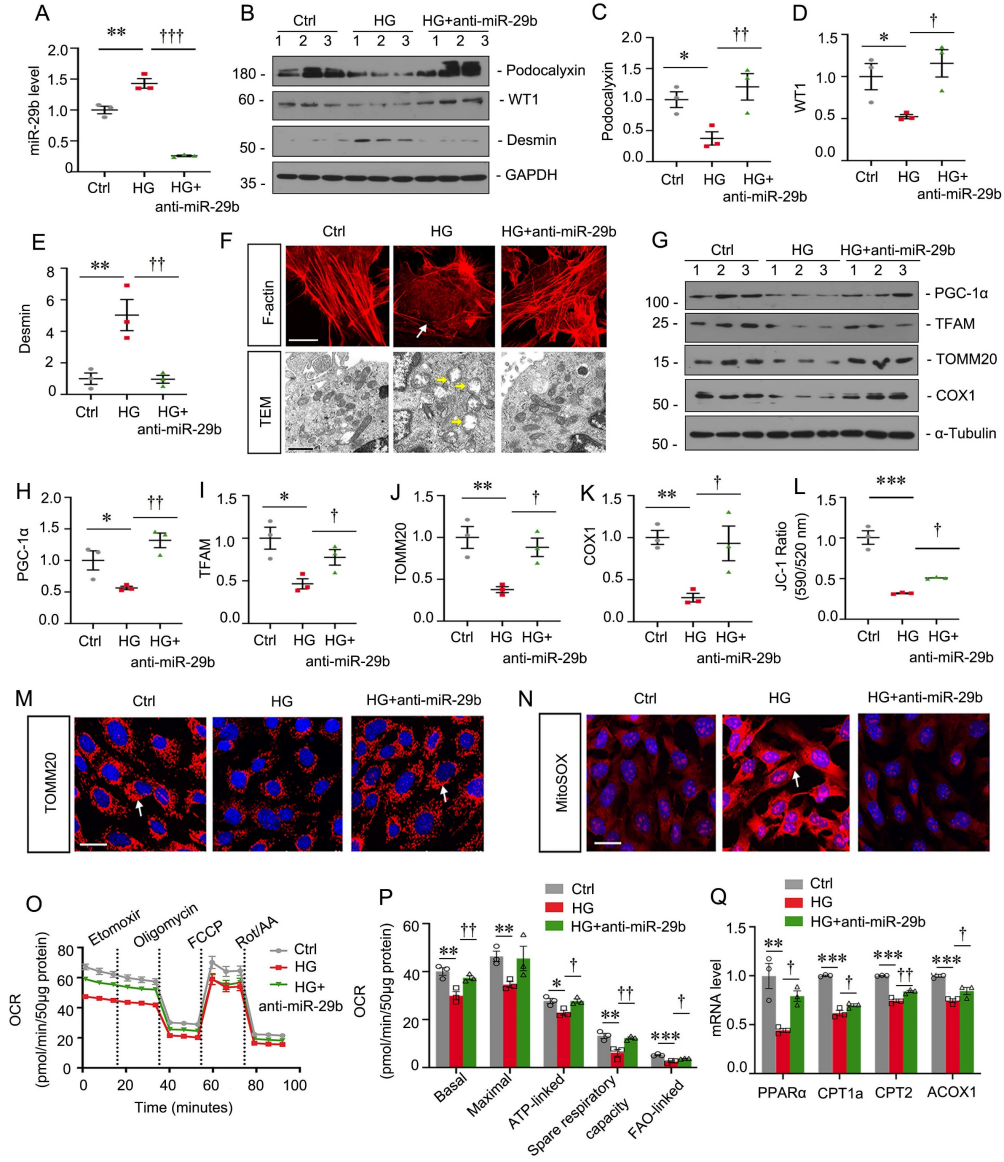
** Inhibition of miR-29b mitigates high glucose-induced podocyte injury by increasing PGC-1α.** (A) qRT-PCR analysis shows the relative levels of miR-29b after high glucose treatment for 48h. **P < 0.01 versus control group (n=3), †††*P* < 0.001 versus high glucose group (n=3). (B-E) Representative western blot and quantitative data showing expression of podocalyxin, WT1 and Desmin. Numbers (1-3) indicate each individual culture in each given group. *P < 0.05, **P < 0.01 versus control group; †*P* < 0.05, ††*P* < 0.01 versus high glucose group (n=3). (F) Representative micrographs showing immunostaining of F-actin. Arrows indicate actin skeleton rearrangement. Bar = 15μm. Representative TEM micrographs showing mitochondrial ultrastructure following different treatments. Arrows indicate injured mitochondria. Bar = 1μm. (G-K) Representative western blot and quantitative data showing expression of PGC-1α, TFAM, TOMM20 and COX1. Numbers (1-3) indicate each individual culture in each given group. *P < 0.05, **P < 0.01 versus control group; †*P* < 0.05, ††*P* < 0.01 versus high glucose group (n=3). (L) Graphical representation of mitochondrial membrane potential (MMP). MMP was detected by JC-1 staining and analyzed by flow cytometry. The data is shown as the ratio of the fluorescence intensity at absorbance of 590 nm (JC-1 aggregate) to 520 nm (JC-1 monomer). ***P < 0.001 versus control group; †*P* < 0.05 versus high glucose group (n=3). (M) Representative micrographs show immunostaining of TOMM20 staining. Arrows indicate positive staining. Bar = 25μm. (N) Representative micrographs show mitoSOX probe staining. Arrows indicate positive staining. Bar = 25μm. (O-P) Graphical representations of basal OCR, maximal OCR, ATP-linked OCR, spare respiratory and FAO-linked OCR in different groups. *P < 0.05, **P < 0.01, ***P < 0.001 versus control group; †*P* < 0.05, ††*P* < 0.01 versus high glucose group (n=3). (Q) Graphical representations of the relative mRNA abundance of PPARα, CPT1a, CPT2 and ACOX1 in different groups. **P < 0.01, ***P < 0.001 versus control group; †*P* < 0.05, ††*P* < 0.01 versus high glucose group (n=3).

MPC5 cells were treated with high glucose and miR-29b inhibitor. As shown in Figure 4A, high glucose significantly induced miR-29b, but co-treatment with miR-29b inhibitor greatly blocked it. High glucose decreased the expression of podocalyxin and WT1, but upregulated Desmin expression. However, co-treatment with miR-29b inhibitor significantly blocked these effects (Figure 4B to E). F-actin staining showed high glucose triggered the actin skeleton rearrangement, but it was blocked by miR-29b inhibitor (Figure 4F). We then examined the ultrastructure of mitochondria by transmission electron microscopy (TEM) assay. As shown in Figure 4F, high glucose induced pronounced changes in podocyte mitochondria with swollen mitochondria and fragmented cristae. However, co-treatment with miR-29b inhibitor largely preserved the normal structure of mitochondria. We next assessed mitochondria-related proteins expression. High glucose significantly decreased the expression of PGC-1α, TFAM, TOMM20 and COX1. However, miR-29b inhibitor significantly restored their expression (Figure 4G to K). JC-1 flow cytometry assay showed high glucose greatly decreased the mitochondrial membrane potential (MMP), while miR-29b inhibitor blocked this (Figure 4L). Furthermore, immunostaining for TOMM20 and MitoSOX staining showed high glucose greatly induced mitochondrial mass loss and mitochondrial ROS production, but co-treatment with miR-29b inhibitor greatly reversed these effects (Figure 4M and N). The OCR assay also showed inhibition to miR-29b greatly reversed high glucose-decreased mitochondrial respiratory function (Figure 4O and P). We then assessed the mRNA levels of PPARα, a key transcriptional factor regulating genes of FAO, and its targets CPT1a, CPT2 and ACOX1. As shown, high glucose treatment decreased their expression, but inhibition to miR-29b preserved their expression (Figure 4Q). These data suggest miR-29b plays an important role in high glucose-induced mitochondrial dysfunction and podocyte injury.

### Inhibition to miR-29b mitigates podocyte injury in ADR nephropathy

**Figure 5:**
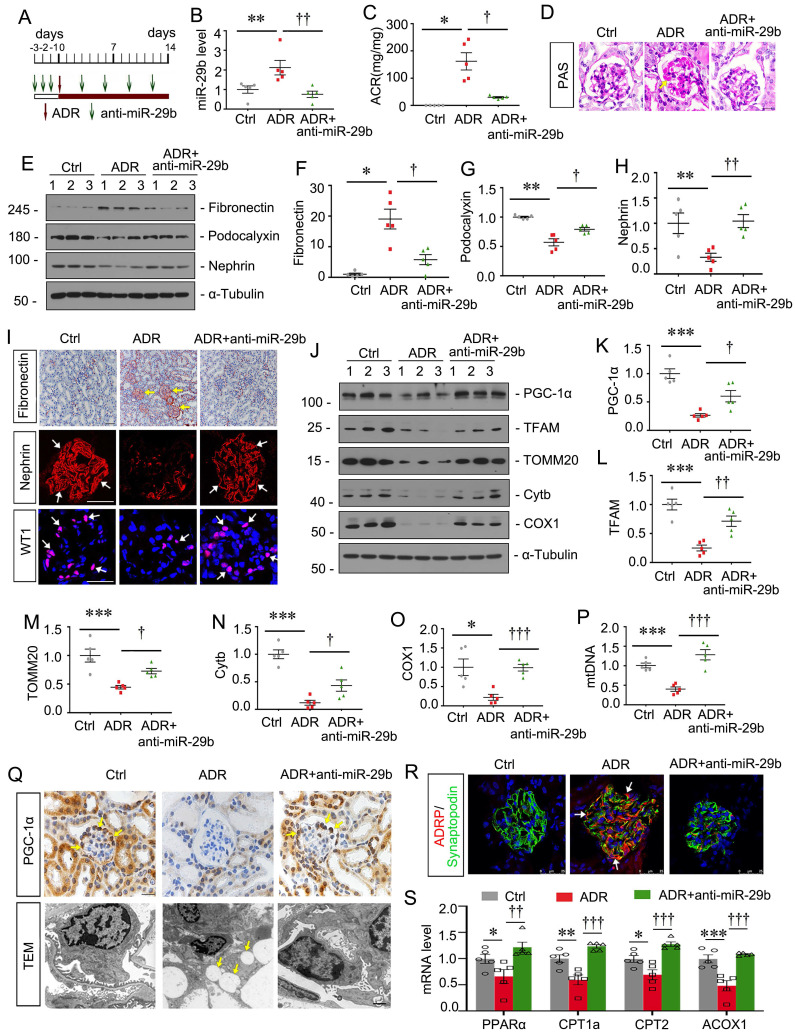
** Inhibition to miR-29b mitigates podocyte injury in ADR nephropathy.** (A) Schematic diagram shows the experimental procedure. (B) qPCR analyses show renal miR-29b level in 3 different groups as indicated. **P < 0.01 versus control group; ††*P* < 0.01 versus ADR group (n=5). (C) Inhibition of miR-29b by antagomiR mitigated proteinuria in ADR mice. Urinary albumin was expressed as mg/mg urinary creatinine. *P < 0.05 versus control group; †*P* < 0.05 versus ADR group (n=5). (D) Representative micrographs show PAS staining in different groups. Arrows indicate positive staining. Bar = 20μm. (E-H) Representative western blot and quantitative data showing expression of fibronectin, podocalyxin and nephrin in different groups. Numbers (1-3) indicate each individual animal in each given group. *P < 0.05, **P < 0.01 versus control group; †*P* < 0.05, ††*P* < 0.01 versus ADR group (n=5). (I) Representative micrographs show immunostaining of fibronectin, nephrin and WT1. Arrows indicate positive staining. Bar = 50μm or 20μm. (J-O) Representative western blot and quantitative data showing expression of PGC-1α, TFAM, TOMM20, Cytb and COX1 in different groups. *P < 0.05, ***P < 0.001 versus control group; †*P* < 0.05, ††*P* < 0.01, †††*P* < 0.001 versus ADR group (n=5). (P) Graph showing the mtDNA level in different groups. ***P < 0.001 versus control group; †††*P* < 0.001 versus ADR group (n=5). (Q) Representative micrographs showing immunostaining of PGC-1α. Arrows indicate positive staining. Bar=20μm. Representative TEM micrographs showing lipid accumulation in ADR group. Bar = 1μm. (R) Co-staining of ADRP (red) and synaptopodin (green) in glomeruli in different groups. Arrows indicate lipid droplets in podocytes. Bar = 25μm. (S) Graphical representations of the relative mRNA abundance of PPARα, CPT1a, CPT2 and ACOX1 in different groups. *P < 0.05, **P < 0.01, ***P < 0.001 versus control group; ††*P* < 0.01, †††*P* < 0.001 versus ADR group (n=5).

To further confirm the effects of miR-29b, we then constructed ADR nephropathy model and co-treated these mice with miR-29b antagomir, the synthetic sequence of anti-miR-29b which was modified with cholesterol at the 3' end, two thioscarbs at the 5' end, four thioscarlets at the 3' end, and a full-chain methoxy modification, to make it more stable and less to be degraded in vivo. The experimental designs are shown in Figure 5A. The inhibition of miR-29b was validated by qPCR (Figure 5B). Furthermore, as shown in Figure 5C, miR-29b antagomiR greatly ameliorated ADR-induced albuminuria. PAS staining showed that miR-29b antagomiR largely ameliorated glomerular sclerotic lesions at 2 weeks after ADR injection (Figure 5D). We then performed western blotting. As shown, miR-29b antagomiR inhibited the protein levels of fibronectin, and restored podocalyxin and nephrin expression in ADR mice (Figure 5E to H). Similar results were observed when fibronectin and nephrin were examined by immunostaining (Figure 5I). We also found miR-29b antagomiR could greatly preserve the expression of WT1, a specific podocyte marker (Figure 5I). We then examined mitochondrial function. As shown, inhibition to miR-29b significantly restored the expression of PGC-1α, TFAM, TOMM20, Cytb and COX1 (Figure 5J to O). Furthermore, we assessed mtDNA content by testing the expression of cytochrome c oxidase subunit 2 (COX2), an important mitochondrially encoded oxidative phosphorylation (OXPHOS) complex IV subunit to transfer electrons from cytochrome c to oxygen. As shown in Figure 5P, miR-29b antagomir significantly reversed the mtDNA level in mice after ADR treatment. PGC-1α was also assessed by immunostaining. As shown in Figure 5Q, PGC-1α was highly expressed in podocytes, but was decreased in ADR mice. miR-29b antagomir could largely reversed its expression. Lipid droplets were also assessed by TEM. As shown, in adriamycin-induced podocytes, there was a great accumulation of lipid droplets, but miR-29b antagomir could strongly inhibit it (Figure 5Q). Co-staining of ADRP, a major component of the lipid droplets, and synaptopodin, a marker of podocyte, showed a high expression of lipid droplet in podocytes in ADR mice, but miR-29b antagomir strikingly blocked it (Figure 5R). We further tested FAO-related genes expression. As shown, mRNA levels of PPARα, CPT1a, CPT2 and ACOX1 were decreased in ADR mice but were preserved by miR-29b antagomir (Figure 5S).

### Ectopic expression of miR-29b aggravates podocyte injury in ADR nephropathy

**Figure 6:**
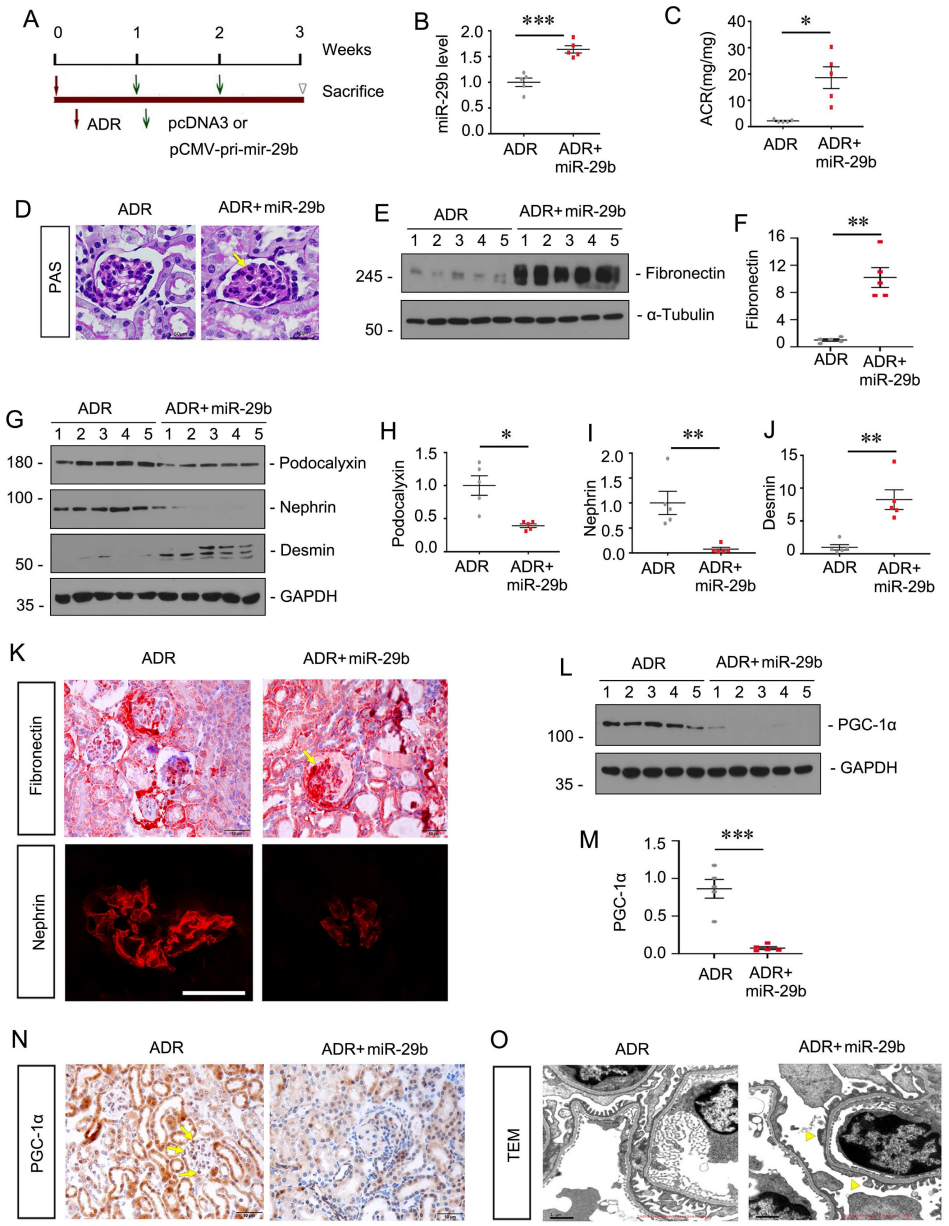
** Ectopic expression of miR-29b aggravates podocyte injury in ADR nephropathy.** (A) Schematic diagram shows the experimental procedure. (B) qPCR analyses show renal miR-29b level in two groups as indicated. ***P < 0.001 versus ADR mice group (n=5). (C) Ectopic expression of miR-29b augmented proteinuria in ADR mice. Urinary albumin was expressed as mg/mg urinary creatinine. *P < 0.05 versus ADR mice group (n=5). (D) Representative micrographs show PAS staining in different groups. Arrows indicate positive staining. Bar = 50μm. (E-J) Representative western blot and quantitative data showing expression of fibronectin, Podocalyxin, nephrin and Desmin in two groups. Numbers (1-5) indicate each individual animal in each given group. *P < 0.05, **P < 0.01 versus ADR mice group (n=5). (K) Representative micrographs show immunostaining of fibronectin and nephrin. Arrows indicate positive staining. Bar = 50μm and 25μm. (L-M) Representative western blot and quantitative data showing expression of PGC-1α in two groups. Numbers (1-5) indicate each individual animal in each given group. ***P < 0.001 versus ADR mice group (n=5). (N) Representative micrographs show immunostaining of PGC-1α. Arrows indicate positive staining. Bar = 50μm. (O) Representative TEM micrographs showing podocytes foot process in two groups. Bar = 1μm.

To explore the role of miR-29b in podocyte injury in vivo, we constructed a mouse model of ADR nephropathy. The experimental design was shown in Figure 6A. The pCMV-pri-mir-29b plasmid was injected intravenously by a hydrodynamic-based gene delivery approach. Mice were sacrificed 3 weeks after ADR injection. As shown in Figure 6B, renal expression of miR-29b was augmented after injections of pri-mir-29b plasmid. As shown in Figure 6C, albuminuria was significantly increased after ectopic expression of miR-29b in ADR mice. PAS staining showed that miR-29b greatly accelerated glomerular sclerotic lesions in ADR mice (Figure 6D). We then examined renal fibrosis and podocyte injury. As shown, ectopic expression of miR-29b increased the protein levels of fibronectin and desmin, and decreased podocalyxin and nephrin expression in ADR mice (Figure 6E and J). Similar results were observed when fibronectin and nephrin were examined by immunostaining (Figure 6K).

We next examined mitochondrial function. Western blot showed the protein level of PGC-1α was significantly decreased after ectopic expression of miR-29b in ADR mice (Figure 6L to M). Similar results were observed when PGC-1α was examined by immunostaining (Figure 6N). We then examined the ultrastructure of podocytes by TEM. As shown in Figure 6O, ectopic expression of miR-29b induced pronounced fusions of foot processes (yellow arrowheads) in podocytes, further suggesting the damaged role of miR-29b in podocyte injury.

### Inhibition to miR-29b mitigates mitochondrial dysfunction and podocyte injury in db/db mice

**Figure 7:**
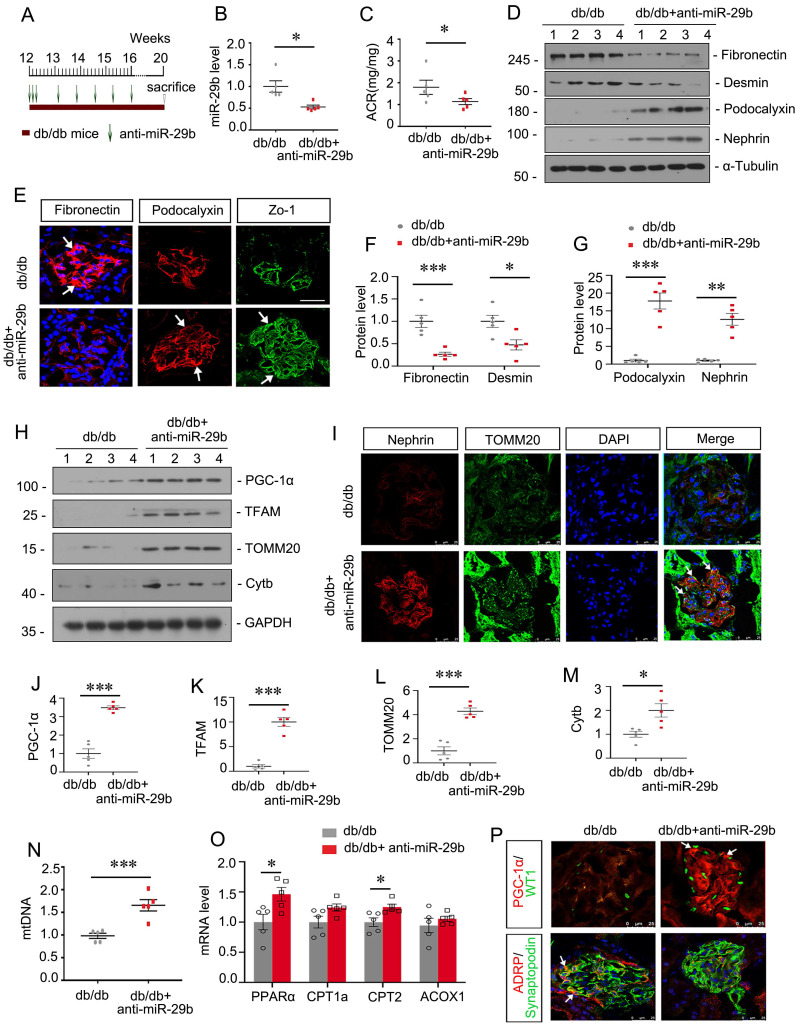
** Inhibition to miR-29b mitigates mitochondrial dysfunction and podocyte injury in db/db mice.** (A) Schematic diagram shows the experimental procedure. (B) qPCR analyses show renal miR-29b level in two groups as indicated. *P < 0.05 versus db/db mice group (n=5). (C) Inhibition of miR-29b by antagomiR mitigated proteinuria in db/db mice. Urinary albumin was expressed as mg/mg urinary creatinine. *P < 0.05 versus db/db mice group (n=5). (D, F-G) Representative western blot and quantitative data showing expression of fibronectin, Desmin, Podocalyxin and nephrin in two groups. Numbers (1-4) indicate each individual animal in each given group. *P < 0.05, **P < 0.01, ***P < 0.001 versus db/db mice group (n=5). (E) Representative micrographs show immunostaining of fibronectin, podocalyxin and Zo-1. Arrows indicate positive staining. Bar = 25μm. (H, J-M) Representative western blot and quantitative data showing expression of PGC-1α, TFAM, TOMM20 and Cytb in two groups. Numbers (1-4) indicate each individual animal in each given group. *P < 0.05, ***P < 0.001 versus db/db mice group (n=5). (I) Co-staining of nephrin and TOMM20 in glomerular podocytes in different groups. Arrows indicate the co-localization of nephrin and TOMM20. Bar = 25μm. (N) Graph showing the mtDNA level in 2 groups. ***P < 0.001 versus db/db mice group (n=5). (O) Graphical representations of the relative mRNA abundance of PPARα, CPT1a, CPT2 and ACOX1 in different groups. *P < 0.05 versus db/db mice group (n=5). (P) Co-staining of PGC-1α (red) and WT1 (green), ADRP (red) and synaptopodin (green) in glomerular podocytes in different groups. Arrows indicate positive staining. Bar = 25μm.

We then assessed the role of miR-29b in db/db mice, a well-established animal model for podocyte injury. The experimental design was shown in Figure 7A. miR-29b antagomir was injected into db/db mice from their age at 12 weeks, an early stage of DKD. The interference efficiency of miR-29b was validated by qPCR (Figure 7B). As shown, miR-29b antagomir greatly decreased albuminuria in db/db mice (Figure 7C). Western blot and immunofluorescence analyses showed miR-29b antagomir significantly inhibited the expression of Fibronectin and Desmin, and restored the expression of Podocalyxin, Nephrin and Zo-1 (Figure 7D to G). We next assessed mitochondria-related proteins. Western blot analyses showed that miR-29b antagomir significantly restored the expression of PGC-1α and its downstream targets, such as TFAM, TOMM20 and Cytb (Figure 7H, J-M). Co-staining of nephrin and TOMM20 showed miR-29b antagomir greatly inhibited podocyte injury and restored mitochondrial mass in podocytes (Figure 6I). Besides, miR-29b antagomir significantly upregulated the mtDNA level, and mRNA levels of PPARα, CPT1a, CPT2 and ACOX1 in db/db mice (Figure 7N and O). Furthermore, co-staining of PGC-1α and WT1, and ADRP and synaptopodin showed miR-29b antagomir greatly upregulated PGC-1α expression and inhibited lipid accumulation in podocytes in db/db mice (Figure 7P).

### Activation of PGC-1α inhibits miR-29b-induced mitochondrial dysfunction and cell injury in podocytes

**Figure 8:**
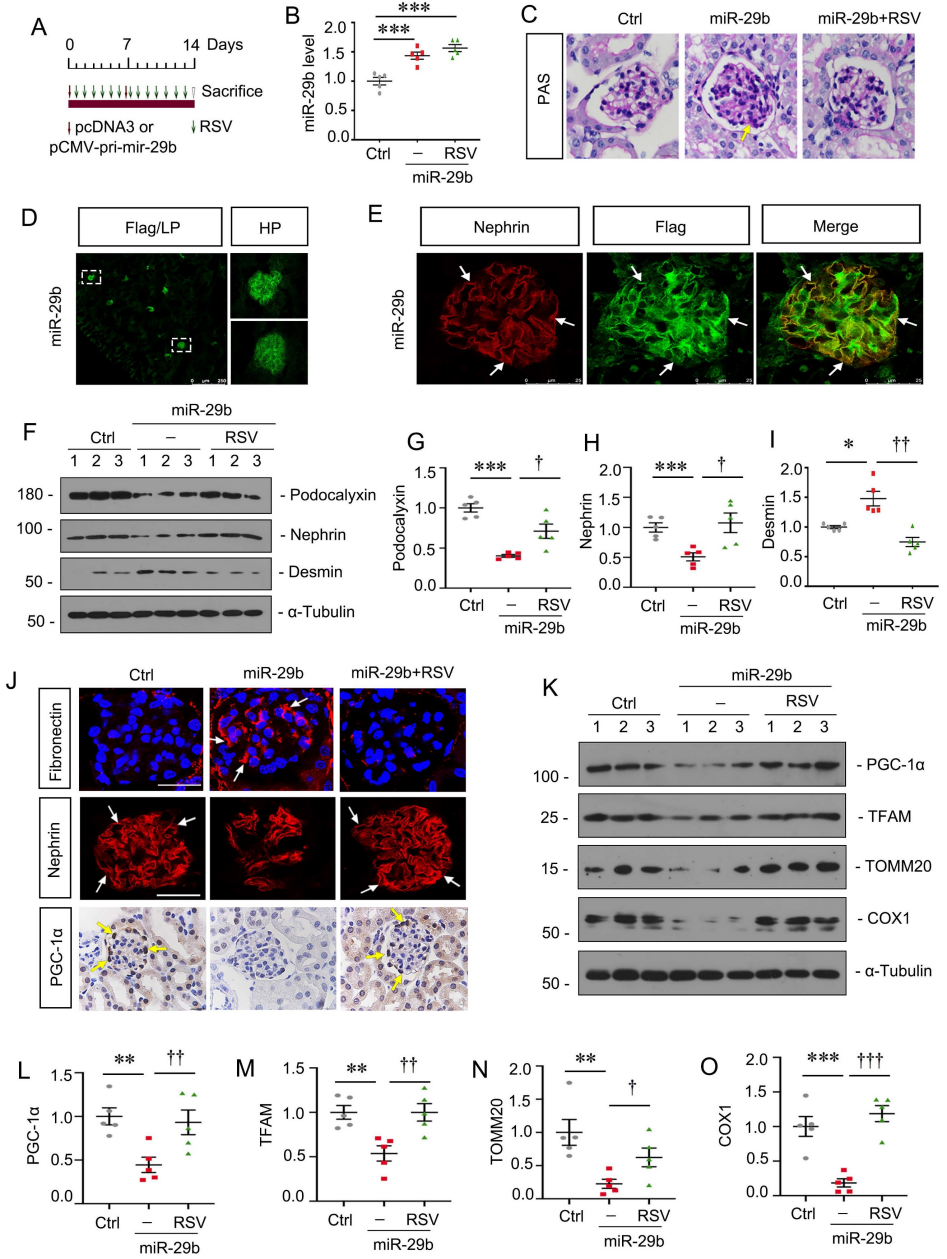
** Activation of PGC-1α inhibits miR-29b-induced mitochondrial dysfunction and cell injury in podocytes.** (A) Schematic diagram shows the experimental procedure. (B) qPCR analyses show renal miR-29b level in different groups as indicated. ***P < 0.001 versus control group (n=5). (C) Representative micrographs show PAS staining in different groups. Arrows indicate positive staining. Bar = 20μm. (D-E) Representative micrographs confirming the specific expression of miR-29b in podocytes through immunofluorescence staining with anti-Flag and nephrin antibodies. Arrows indicate positive staining. Bar = 250μm or 25μm. (F-I) Representative western blot and quantitative data showing expression of podocalyxin, nephrin and Desmin in different groups. Numbers (1-3) indicate each individual animal in each given group. *P < 0.05, ***P < 0.001 versus control group; †*P* < 0.05, ††*P* < 0.01, †††*P* < 0.001 versus miR-29b plasmid group (n=5). (J) Representative micrographs show immunostaining of fibronectin, nephrin and PGC-1α. Arrows indicate positive staining. Bar = 20μm or 50 μm. (K-O) Representative western blot and quantitative data showing expression of PGC-1α, TFAM, TOMM20 and COX1 in different groups. **P < 0.01, ***P < 0.001 versus control group; †*P* < 0.05, ††*P* < 0.01, †††*P* < 0.001 versus miR-29b plasmid group (n=5).

To further confirm the role of miR-29b in regulating of PGC-1α, mice were injected with miR-29b expressing plasmid and co-treated them with Resveratrol (RSV), an activator of PGC-1α (Figure 8A). In vivo expression of miR-29b was validated by qPCR (Figure 8B). As shown, RSV largely ameliorated glomerular sclerotic lesions in miR-29b-overexpressed mice (Figure 8C). Overexpression of miR-29b was observed in glomeruli through detection of Flag-labeled fluorescence (Figure 8D). Co-staining of Flag-labeled miR-29b and nephrin showed miR-29b was localized in podocytes (Figure 8E). We then assessed podocyte injury. As shown in Figure 8F to I, administration of RSV significantly restored the expression of podocalyxin and nephrin and inhibited the expression of Desmin. Similar result can be observed when fibronectin and nephrin were assessed by immunostaining (Figure 8J). We then assessed mitochondrial function. As shown in Figure 8J, RSV greatly restored the expression of PGC-1α in podocytes after delivery of pri-mir-29b plasmid. Western blot analyses showed PGC-1α, TFAM, TOMM20 and COX1 were also preserved by RSV in miR-29b-overexpressed mice (Figure 8K to O). These data further confirmed miR-29b induced mitochondrial dysfunction and cell injury in podocytes through targeting PGC-1α.

### Specific ablation of miR-29b in podocytes mitigates high glucose-induced podocyte injury and mitochondrial dysfunction in glomerular mini-organ culture

**Figure 9:**
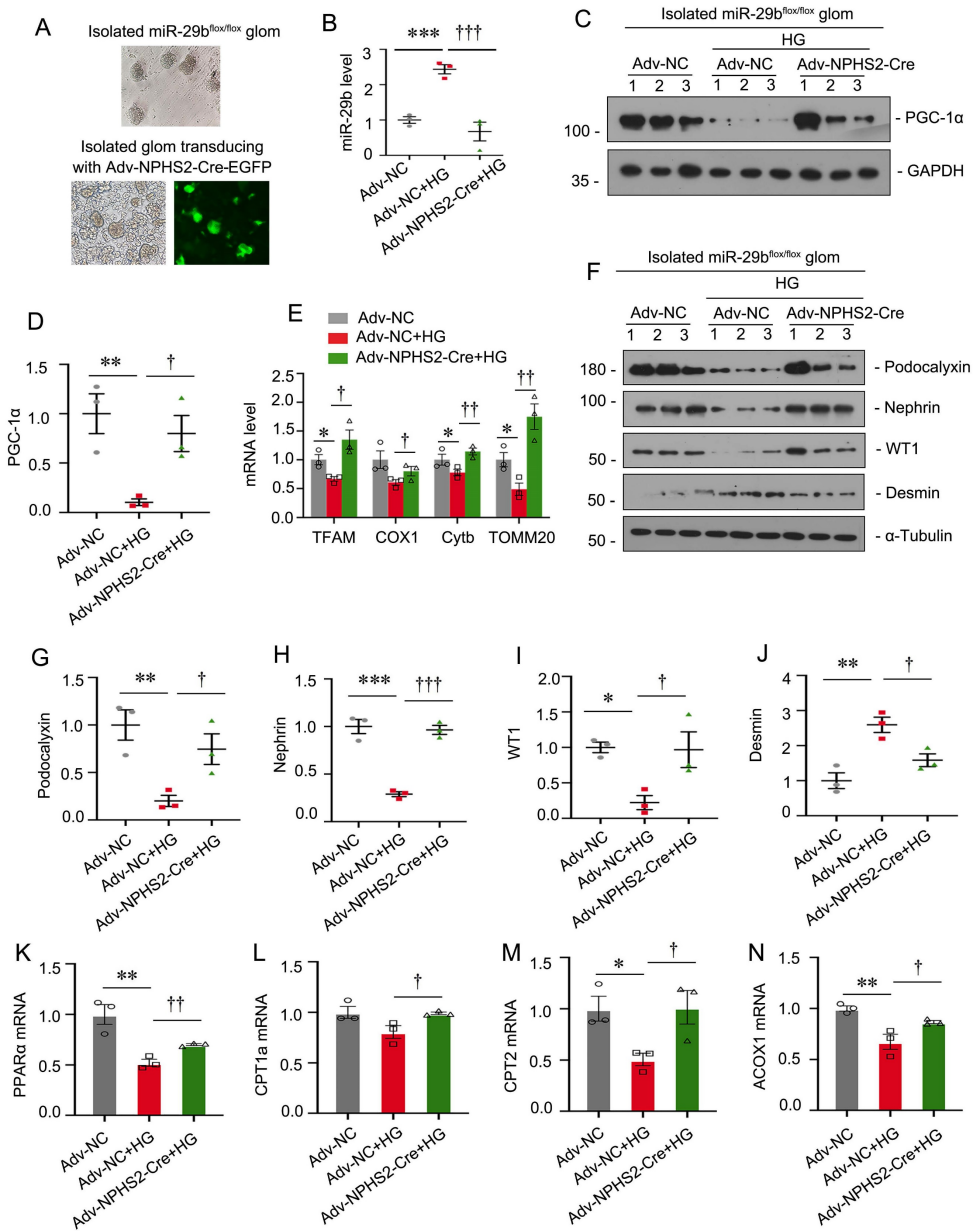
** Specific ablation of miR-29b in podocytes mitigates high glucose-induced podocyte injury and mitochondrial dysfunction in glomerular mini-organ culture.** (A) Representative image showing the isolated miR-29b^flox/flox^ mice glomeruli under microscopy. (B) Graph showing the miR-29b level in Adv-NC group, Adv-NC+HG group and Adv-NPHS2-Cre+HG group. ***P<0.001 versus negative control group (Adv-NC); †††*P*<0.001 versus high glucose group (Adv-NC+HG) (n=3). (C-D) Representative western blot and quantitative data showing expression of PGC-1α. Numbers (1-3) indicate each individual culture in each given group. **P < 0.01 versus negative control group (Adv-NC); †*P* < 0.05 versus high glucose group (Adv-NC+HG) (n=3). (E) Graphical representations of the relative mRNA abundance of TFAM, COX1, Cytb and TOMM20 in different groups. *P < 0.05 versus negative control group (Adv-NC); †*P* < 0.05, ††*P* < 0.01 versus high glucose group (Adv-NC+HG) (n=3). (F-J) Representative western blot and quantitative data showing expression of podocalyxin, nephrin, WT1 and Desmin. Numbers (1-3) indicate each individual culture in each given group. *P < 0.05, **P < 0.01, ***P < 0.001 versus negative control group (Adv-NC); †*P* < 0.05, ††*P* < 0.01 versus high glucose group (Adv-NC+HG) (n=3). (K-N) Graphical representations of the relative mRNA abundance of PPARα, CPT1a, CPT2 and ACOX1 in different groups. *P < 0.05, **P < 0.01 versus negative control group (Adv-NC); †*P* < 0.05, ††*P* < 0.01 versus high glucose group (Adv-NC+HG) (n=3).

To further demonstrate the role of miR-29b in podocyte injury, we established miR-29b^flox/flox^ mice and isolated glomeruli for mini-organ culture (Figure 9A). The specific ablation of miR-29b in podocytes was achieved by transducing isolated glomeruli from miR-29b^flox/flox^ mice with adenovirus encoding NPHS2-driving Cre recombinase (Figure 9A-B). Glomerular mini-organ culture was treated with high glucose. As shown in Figure 9C to E, specific ablation of miR-29b in podocytes could significantly restore the expression of PGC-1α, as well as mitochondria -related genes such as TFAM, COX1, Cytb and TOMM20. Besides, knockout of miR-29b in podocytes significantly preserved the expression of podocalyxin, nephrin, WT1 and inhibited the expression of Desmin, as assessed by western blot analyses (Figure 9F to J). Furthermore, we assessed FAO-associated genes. As shown, knockout of miR-29b in podocytes significantly restored PPARα, CPT1a, CPT2 and ACOX1 expression (Figure 9K to N). These data further suggest miR-29b plays a key role in mitochondrial dysfunction and cell injury in podocytes.

**Figure 10:**
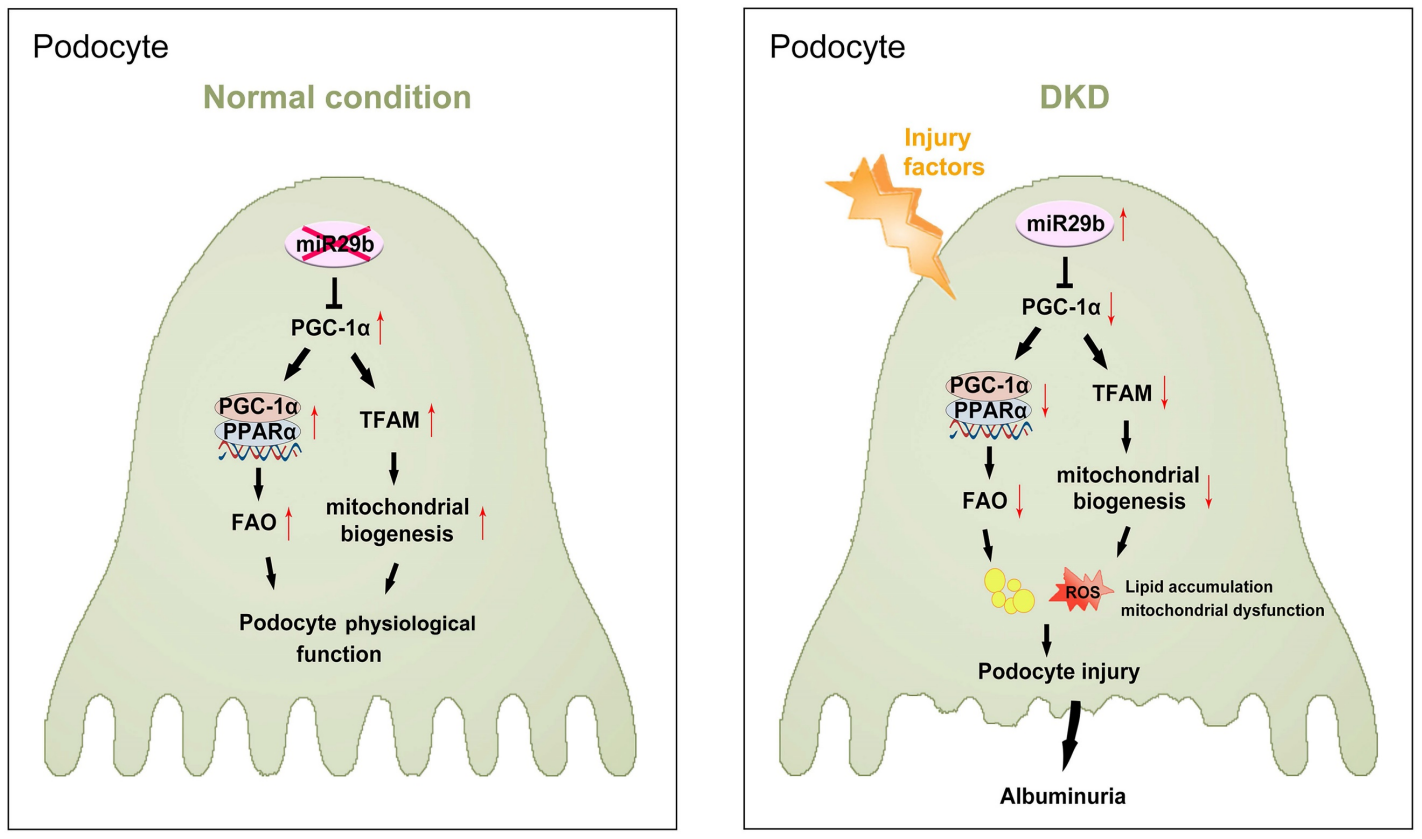
**The schematic presentation depicts the potential mechanism that miR-29b targets PGC-1α and promotes mitochondria dysfunction and podocyte injury in DKD.** The schematic presentation depicts the potential mechanism by which miR-29b promotes podocyte injury and albuminuria in DKD. MiR-29b may target PGC-1α and then inhibit the activity of fatty acid oxidation and mitochondrial biogenesis, eventually leading to lipid accumulation and mitochondrial dysfunction in podocyte. These effects lead to podocyte injury, which further induces albuminuria and DKD progression.

Hence, as summarized in Figure 10, we concluded miR-29b plays an important role in podocyte injury and DKD progression. Through targeting PGC-1α, miR-29b inhibits the activity of FAO and mitochondrial biogenesis in podocytes. This leads to lipid accumulation and loss of mitochondrial homeostasis, which collectively promotes podocyte injury and albuminuria.

## Discussion

miR-29s are highly involved in cell proliferation, differentiation, angiogenesis, apoptosis, especially in tumour cells. miR-29 could also be a carcinogenic molecule or a tumour suppressor molecule^27^-^29^. However, miR-29s also possess the ability of anti-fibrosis. miR-29s could target multiple collagen genes (11 out of 20), as well as various extracellular matrix-related genes such as MMP2, FBN1 and ITGB1. Studies have already confirmed the anti-fibrotic effects of miR-29 in heart, lung, liver, skin and kidney^24^-^26^, ^30^, ^31^. In kidney disease, studies showed that miR-29 is downregulated in renal tubular epithelial cells and plays an antifibrotic effect through targeting collagens^26^, ^32^. But miR-29 was upregulated in podocytes to trigger albuminuria and glomerular sclerosis^33^. Consistently, we found miR-29b was upregulated in podocytes but downregulated in renal tubular cells (HKC8), human umbilical vein endothelial cells (HUVECs) and rat mesangial cell line (HBZY-1) after treatment with high glucose (Figure S2 A-D). Furthermore, we isolated glomeruli and tubules, and found increased miR-29b in glomeruli and significantly unchanged miR-29b expression in tubules in db/db mice at 20 weeks of age. In mice with 2 weeks of ADR injection, we found increased miR-29b expression in glomeruli but decreased expression of miR-29b in tubules (Figure S2 I-J, Figure 1A-C). These suggest miR-29s exert different functions in different types of renal cells. To clarify the specific role of miR-29s family members in podocyte injury, we measured miR-29a, miR-29b and miR-29c expression in isolated glomeruli. Results showed miR-29b expression was significantly upregulated in the glomeruli from db/db and ADR mice. We also found miR-29b increased in podocytes in ADR mice. These suggest miR-29b could play a key role in podocyte and glomerular injury.

Several lines of evidences supported the pathologic role of miR-29b in diabetic kidney disease (DKD). First, miR-29b was the most upregulated miR-29s family member in glomeruli of db/db mice, and significantly increased in the urine of patients with early DKD (stages I and II). Urinary miR-29b was negatively correlated with eGFR, and positively correlated with nephrin in urine. Second, miR-29b overexpression impaired podocyte integrity in vitro and in vivo. Interestingly, we found the target of miR-29b is PGC-1α, a key transcription factor regulating mitochondrial biogenesis. Hence, we also found urinary miR-29b was positively correlated with mtDNA content in urine. Ectopic expression of miR-29b triggered mitochondrial loss, mitochondrial ROS production, and respiratory ability defects, and FAO functional disorder in podocytes. Activation of PGC-1α could resist miR-29b-induced podocyte injury and mitochondrial dysfunction. Third, interference of miR-29b in diabetic nephropathy mice effectively blocked podocyte injury and restored mitochondrial function in podocytes. Of note, knockout of miR-29b specifically in podocytes could greatly restore the expression of PGC-1α, mitochondrial function, and podocyte integrity. Therefore, our research sufficiently implicates miR-29b plays a key role in podocyte injury and diabetic nephropathy through inducing mitochondrial dysfunction.

The regulations of microRNAs on targeted mRNAs are quite intricate. Each microRNA may regulate hundreds of genes expression in different spatial and temporal contexts, and each gene may also be regulated by hundreds of microRNAs^34^, ^35^. In addition to PGC-1α, we found that PPARD, CREB5, and ATP5G1 are also predicted targeted genes of miR-29b. PPARδ, encoded by the PPARD gene, is a member of the family of transcriptional regulator PPAR, and regulates fatty acid oxidation process in mitochondria with the synergistic effect of PGC-1α^36^. The cAMP response element binding protein encoded by the CREB5 gene could activate the transcriptional activity of PGC-1α, which regulates PGC-1α at gene level, differing from the post-transcriptional regulation of PGC-1α by miR-29b^37^. The ATP5G1 gene encodes ATP synthase on the mitochondrial respiratory chain complex F0, regulating mitochondrial biogenesis and oxidative phosphorylation activities. These results suggest miR-29b regulates mitochondrial function not only by targeting PGC-1α, but also regulating multiple PGC-1α-related genes, to better modulate mitochondrial biogenesis and maintain mitochondrial function in multiple directions.

The effective control of PGC-1α is important for the stability of mitochondrial homeostasis. Studies showed excessive PGC-1α in heart would lead to the fragmentation of mitochondria, defective oxidative phosphorylation and ROS production, resulting in cardiometabolic disorders and the tendency to heart failure and premature death^38^. In kidney disease, studies found that overexpression of PGC-1α is beneficial for renal tubular cells in acute kidney injury model (AKI)^39^, ^40^. However, overexpression of PGC-1α in glomerular podocytes leads to collapsing glomerulopathy. These suggest that overactivation of PGC-1α is pathogenic to podocytes and glomerulus, and there is a narrow window for therapeutic levels of PGC1α in podocytes^41^. In this study, we found PGC-1α was significantly downregulated in podocytes in DKD model, resulting in mitochondrial dysfunction, podocyte cell damage and glomerulosclerosis. However, antimiR-29b could restore PGC-1α, and ameliorate podocyte injury and renal fibrosis, suggesting antimiR-29b could regulate PGC-1α levels at a normal or suitable level. As excessive PGC-1α inducing podocyte injury, to inhibit miR-29b may be a safe and an effective approach to protect podocyte integrity through upregulating PGC-1α at a suitable therapeutic window.

Our study also has significant clinical implications. We found miR-29b and mtDNA levels were induced in the urine in patients with diabetic kidney disease at 1-2 stage, which is consistent with some previous studies 42-44. We further found the increased miR-29b levels in urine were negatively correlated with eGFR, and positively correlated with urinary mtDNA and nephrin content. These correlations provide further evidence that miR-29b mediates podocytes injury by targeting inhibition on mitochondrial function. Moreover, miR-29b can be used as a new marker for diagnosis of early diabetic nephropathy, as well as predicting disease progression. However, the change of miR-29b in urine is not consistent in all chronic kidney diseases. Wang.G found that urinary miR-29b levels in patients with IgA nephropathy were significantly downregulated than those in healthy controls, and were negatively correlated with proteinuria levels and positively correlated with eGFR 45, which may be due to different pathogenesis of DKD from IgA nephropathy and different stages of CKD in enrolled patients. Therefore, urinary miR-29b may have disease specificity to be a diagnostic marker. In this study, we demonstrated urinary miR-29b can be a specific marker for diagnosing early diabetic nephropathy, and miR-29b inhibitor was an effective therapeutic strategy.

However, some of our results are not consistent with the previous ones. Several previous studies found that miR29b has an anti-renal fibrosis effect, and overexpression of miR29b not only reduces glomerular, cortical, and medullary fibrosis in db/db but also reduces the protein/creatinine ratio in db/db 46-48. Some studies concluded that miR29b expression in 20-week-old db/db mouse kidneys decreased by 60% compared to 20-week-old db/m mouse kidneys and this led to a significant increase in db/db proteinuria and overexpression of miR29b significantly improved db/db proteinuria levels 48. In our study, we analyzed the expression of miR-29b in db/db mice at 8, 12, 20, and 40 weeks of age. The results showed there was no difference of miR-29b expression in db/db mice at 8, 12, and 20 weeks of age, compared to db/m controls. However, there was a strong reduction of miR-29b in db/db mice at 40 weeks of age (Figure S2 E-H). Hence, we concluded miR-29b indeed lost its expression in the late stage of DN. As for why our results showed no difference of miR-29b in db/db mice at 20 weeks of age, we thought this was because the db/db mice were bought form different companies, and their living environment would inevitably lead to the discrepancy of disease progression. However, we have assessed the expression of miR-29b in db/db mice at 40 weeks of age, and found it was strongly reduced, further supportive to the previous studies showing its decreased trend in late stage of DN.

About the discrepancy of therapeutic effects of miR-29b in db/db mice, we thought there were several reasons: i. Indeed, the previous study showed ectopic miR-29b ameliorated renal fibrosis in both db/db and UUO mice. From the ISH staining of miR-29b, we could found a high abundance of miR-29b was located in tubules 26, 48 and maybe mesangium to some extent (only from our visual observation) after miR-29b expressing plasmid transferring. We thought this was because the approach of the study, ultrasound-mediated gene transfer of large amounts of DNA (100~200μg) into kidney, was prone to mediate a large amount of DNA into some types of vulnerable cells surrounding with abundant blood vessels, like tubular cells or mesangial cells. Furthermore, from the previous studies, we found high glucose decreased miR-29b expression in mesangial cells, and TGF-β triggered a decrease in miR-29b in tubular cells. As miR-29b targeted against collagen in these cells, we thought the supplementation of miR-29b could protect against renal fibrosis through targeting against some fibrogenesis-related genes. Indeed, the previous studies showed ectopic miR-29b decreased fibronectin, collagen I, III and IV, which blocked the disease progression in both DN and UUO mice. These led to a reduction of proteinuria to some extent, in DN mice, even if it was still significantly higher than control mice. ii. In our study, we injected miR-29b expressing plasmid (~20 μg) into the tail vein of mice through hydrodynamic-based gene delivery approach, which mainly located in glomeruli (Fig. 8D) in kidney. To testify the expressional location of ectopic miR-29b, we labeled this expressing plasmid with Flag-tag. As shown in Fig. 8E, Flag-tagged miR-29b was largely co-localized with nephrin, a special marker of podocyte, although some seemed to deposit in mesangial zone. We then tested podocyte markers and found ectopic miR-29b decreased PGC-1α expression in podocytes and induced podocyte injury. Hence, we thought miR-29b could inhibit different targets in different cells. iii. To test whether miR-29b plays a role in podocyte injury, the gene ablation of it in special cell is of great importance. We constructed miR-29b ^flox/flox^ mice to isolate glomeruli and transferred NPHS2-cre adenovirus to knockout miR-29b specifically in podocytes. In this mini-organ culture system, we found podocyte-specific knockout of miR-29b significantly restored PGC-1α, TFAM, COX1, Cytb, and TOMM20, preserved PPARα, CPT1, CPT2, and Acox1, and restored podocalyxin, nephrin, and WT1. These results clearly showed podocyte miR-29b damaged mitochondrial function and triggered cell injury. Collectively, we had sufficiently demonstrated podocyte miR-29b plays a key role in mitochondrial injury and cell injury. Consistently, we found miR-29b antagomiR restored podocyte integrity in ADR or db/db mice, and overexpression of miR-29b accelerated podocyte injury in ADR mice and even in control mice. Of interest, we also observed beneficial effects of TOMM20 or PGC-1α expression in tubules in anti-miR-29b-treated db/db or ADR mice. As we thought, the reason behind lies in the improvement of glomerular injury. As previous studies had shown, glomerular injury could lead to the connected renal tubules injury 49-51. Furthermore, miR-29b did not induce tubular cell injury, and the correlation analysis also showed there was no correlation between miR-29b and N-Gal, a tubular cell injury marker. Hence, we thought the upregulation of TOMM20 or PGC-1α came from the improvement of glomeruli.

In summary, our results showed the role of miR-29b in podocytes is differing from its anti-fibrotic effects in fibroblasts and tubular cells. We demonstrated that miR-29b mediates podocyte injury and DKD progression by targeting PGC-1α and inducing mitochondrial dysfunction. At the early stages of DKD, we found the expression of miR-29b in renal tubular cells was not changed, but it was significantly upregulated in podocyte to induce cell damage. With the progression of DKD, miR-29b expression in tubular cells was significantly downregulated, resulting in increased expression of collagen to aggravate renal fibrosis. The early using of miR-29b inhibitor can slow the progression of DKD. In addition, urinary miR-29b is a specific diagnostic biomarker for the early stages of diabetic nephropathy. Our study is the first to provide a new insight of pathologic role of miR-29b in podocyte injury and provides a new strategy for early intervention on podocyte injury.

## Supplementary Material

Supplementary figures and table.

## Abbreviations

AKI: acute kidney injury
ADR: adriamycin nephropathy
COX1: complex IV subunits cytochrome c oxidase 1
COX2: cytochrome c oxidase subunit 2
DKD: diabetic kidney disease
ESRD: end-stage renal disease
eGFR: estimated glomerular filtration rate
FAO: fatty acid β-oxidation
ISH: in situ hybridization
miRNAs: MicroRNAs
MMP: mitochondrial membrane potential
OXPHOS: oxidative phosphorylation
OCR: oxygen consumption rate
PGC-1α: proliferator-activated receptor-γ coactivator-1α
Prx3: peroxide reductase 3
RSV: Resveratrol
TEM: transmission electron microscopy
UTR: untranslated regions
