# Supplementary material for: MicroRNA-29b Plays a Vital Role in Podocyte Injury and Glomerular Diseases through Inducing Mitochondrial Dysfunction
Source: Int J Biol Sci. 2024 Sep 3;20(12):4654–73. doi: 10.7150/ijbs.93506 (PMC11414390; doi:10.7150/ijbs.93506)
Supplement: Supplementary file 1 — Supplementary figures and table. [file ijbsv20p4654s1.pdf]

Supplementary Table S1. Clinical sample source: Urine samples from healthy individuals and DKD patients

Healthy individuals

No.	Age
1	48
2	48
3	66
4	56
5	61
6	47
7	50
8	50
9	58
10	48
11	51
12	57
13	62
14	56
15	54
16	56
17	66
18	60

DKD patients

No.	Age	gender	DKD Stage	ACR(mg/g)	eGFR(ml/min/1.73m^2)
1	45	male	1	59.84124621	118.75
2	32	female	1	5563.495391	104.14
3	53	female	1	51.45498783	105.42
4	47	male	1	60.79779917	110.84
5	58	female	1	95.87852495	102.44
6	58	female	1	9.724149602	108.49
7	38	male	1	51.26542751	107.25
8	19	female	1	37.41798942	157.68
9	60	male	1	353.3763441	99.89
10	63	male	1	373.6875522	102.42
11	71	female	1	22.58559019	95.43
12	32	male	1	559.0298851	124.77
13	58	male	1	1144.113031	95.09
14	40	male	1	5.78145154	129.93
15	62	female	1	53.21535893	97.11
16	63	female	1	47.9758794	99.57
17	58	female	1	23.33836207	99.28
18	56	male	1	795.3450817	99.13
19	25	female	1	95.88256149	120.14

20	34	female	1	173.0677006	123.75
21	52	female	1	165.784	108.68
22	43	male	1	22.29780309	105.03
23	64	female	1	335.4083601	91.91
24	57	male	1	9.07429533	99.3
25	62	female	1	33.19761954	101.19
26	67	male	1	2822.181232	92.29
27	65	male	1	214.6680896	93.06
28	27	male	1	12.38547124	132.82
29	55	male	1	671.4268143	106.15
30	49	male	1	31.63112727	116.31
31	42	female	1	15.90056589	120.19
32	54	female	1	15.64412121	108.06
33	52	male	1	588.8176409	105.67
34	62	male	1	99.21463681	115.61
35	46	male	1	9.800915883	125.63
36	47	female	1	9.698299506	122.2
37	49	female	1	705.2835314	106.4
38	47	male	1	553.4866071	102.72
39	76	female	1	28.4048048	98.81
40	48	male	1	54.11799556	110.06
41	64	female	1	302.0970532	92.5
42	58	female	1	63.42464813	99.28
43	62	female	1	180.3113946	102.37
44	57	male	1	37.0619883	99.58
45	47	female	1	39.91530612	109.95
46	57	female	1	49.28059464	106.03
47	39	male	1	326.8108856	124.78
48	69	male	1	594.6564605	106.7
49	51	female	1	43.70036618	92.8
50	65	female	1	36.87661575	92.88
51	61	male	1	14.71633427	96.31
52	64	male	1	2526.018589	90.68
53	52	male	1	3127.020099	109.29
54	39	male	1	10426.74714	117.24
55	71	male	1	34.09918963	92.29
56	23	male	1	131.7597772	133.8
57	46	male	1	604.9795845	96.97
58	62	female	1	429.6927083	92.64
59	67	female	1	939.2245274	91.07
60	49	female	1	81.65461847	117.44
61	46	female	1	37.20754717	116.59
62	63	male	1	3359.460575	99.7
63	44	male	1	545.589182	110.41

64	65	male	2	627.7007683	81.29
65	73	female	2	5.966925413	73.29
66	68	male	2	308.8725537	84.07
67	51	male	2	53.25663717	84.9
68	69	male	2	654.9761411	77.57
69	66	female	2	285.9498392	86.68
70	53	male	2	6.976578411	67.91
71	65	female	2	743.6	67.67
72	46	male	2	341.5727891	75.58
73	48	male	2	1973.317133	78.22
74	56	female	2	323.2421895	62.42
75	74	female	2	11.4687219	60.73
76	78	female	2	5.5260466	64.19
77	61	female	2	12.06661207	85.94
78	66	female	2	1015.027098	65.21
79	65	male	2	446.8512195	64.61
80	69	male	2	64.19238477	78.32
81	72	male	2	796.8995934	71.89
82	81	female	2	28.33417421	75.16
83	58	male	2	6367.746667	78.7
84	68	female	2	24.19462747	80.87
85	62	female	2	825.9040853	63.26
86	89	male	2	156.3222116	79.07
87	78	male	2	128.757245	84.93
88	78	male	2	103.3301527	60.37
89	66	male	2	220.0167495	80.72
90	71	male	2	98.66713851	64.95
91	81	male	2	124.3720101	84.13
92	66	male	2	56.51378299	62.69
93	63	male	2	1100.59083	75.99
94	83	male	2	79.18491357	86.63
95	84	female	2	756.0961025	81.27
96	61	female	2	29.40410474	69.6
97	38	male	2	18.58761129	60.42
98	71	male	2	88.5657648	74.77
99	73	male	2	645.9535247	82.62
100	58	male	2	159.8858551	78.7
101	70	male	2	3954.671119	67.02
102	72	female	2	405.9825949	87.2
103	69	male	2	2803.601812	69.2
104	41	male	2	2360.839549	61.82
105	64	male	2	28.70221232	84.23
106	69	male	2	399.4724324	86.26
107	90	female	2	45.56381487	61.65

108	77	female	2	48.86227545	89.06
109	58	male	2	81.25239617	87.85
110	72	female	2	818.833707	66.56
111	87	female	2	4242.348977	60.76
112	59	female	2	147.195122	87.73
113	62	female	2	91.32012012	60.66
114	83	male	2	636.4327694	78.85
115	65	male	2	53.10372447	82.45
116	55	male	2	22.18680514	79.34
117	33	male	2	5752.885757	85.85
118	72	male	2	63.84654951	67.75
119	88	male	2	355.0072359	79.68
120	66	male	2	248.1962306	76.41
121	62	female	2	93.67647059	70.19
122	49	female	2	870.5390909	79.35
123	72	male	2	30.27261518	72.28
124	87	female	2	20.46998285	69.51
125	73	male	2	20.63257257	65.62
126	62	male	2	292.9368145	88.99
127	59	male	3	2444.58677	35.92
128	74	female	3	291.5146699	45.56
129	77	female	3	404.6668017	51.07
130	69	male	3	17.56619088	58.06
131	78	male	3	17.91063003	46.1
132	68	male	3	599.0621153	31.95
133	53	male	3	1001.849771	39.08
134	56	female	3	26.717477	46.27
135	67	female	3	1387.593781	34
136	63	male	3	2155.318735	47.37
137	83	female	3	685.9895452	55.37
138	67	female	3	905.3714286	54.08
139	78	female	3	1093.162767	31.47
140	48	male	3	4181.835893	53.59
141	66	female	3	158.2487429	54.46
142	82	female	3	78.2300885	38.96
143	68	female	3	1944.320578	42.99
144	88	female	3	33.28159816	58.47
145	74	female	3	811.6611391	35.78
146	66	male	3	1063.512498	45.38
147	57	male	3	949.4814815	51.23
148	54	male	3	524.1738579	48.41
149	53	male	3	8.454571027	39.08
150	52	male	3	1244.808037	58.94
151	68	female	3	27.5431706	48.94

152	83	male	3	11.77545195	45.8
153	66	male	3	589.3333333	57.41
154	40	male	3	1792.943446	51.1
155	59	male	3	3545.024691	55.39
156	65	male	3	13.67741935	59.07
157	74	male	3	46.16897081	51.53
158	61	female	3	563.8581673	52.34
159	66	female	3	96.85186705	53.09
160	35	male	3	112.473863	58.08
161	49	female	3	101.2446931	58.35
162	37	male	3	208.2315597	57.42
163	51	female	4	4102.081942	26.52
164	60	male	4	1800.473029	25.9
165	73	female	4	4461.102012	17.81
166	64	female	4	2067.882006	22.04
167	64	male	4	469.4497496	23.91
168	72	female	4	1426.576637	26.25
169	73	female	4	3848.622416	17.81
170	74	female	4	1333.424412	20.32
171	30	male	4	1059.959019	19.4
172	81	female	4	832.0747007	23.12
173	65	female	5	2760.131798	7.64
174	74	female	5	8981.066707	11.08
175	82	female	5	6630.687403	5.03
176	69	female	5	461.9370213	12.62
177	59	female	5	1166.133102	2.84
178	71	female	5	2225.952092	10.16

Supplementary Figures

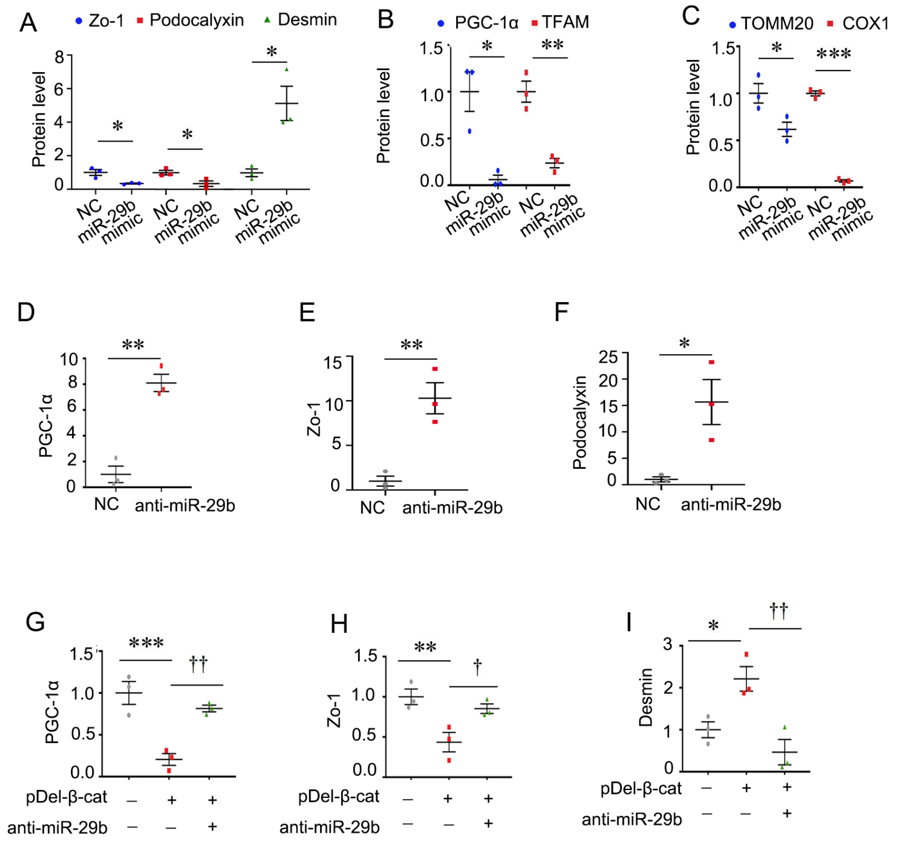

Figure S1

Supplementary Figure S1. MiR-29b promotes podocyte injury by targeting PGC-1α in vitro

(A-C) Quantitative data for Figure 3F and Figure 3G. **P* < 0.05, ***P* < 0.01, ****P* < 0.001 versus control group (n=3). (D-F) Quantitative data for Figure 3L. **P* < 0.05, ***P* < 0.01 versus control group (n=3). (G-I) Quantitative data for Figure 3M. **P* < 0.05, ***P* < 0.01, ****P* < 0.001versus control group; †*P* < 0.05, ††*P* < 0.01 versus pDel-β-catenin group (n=3).

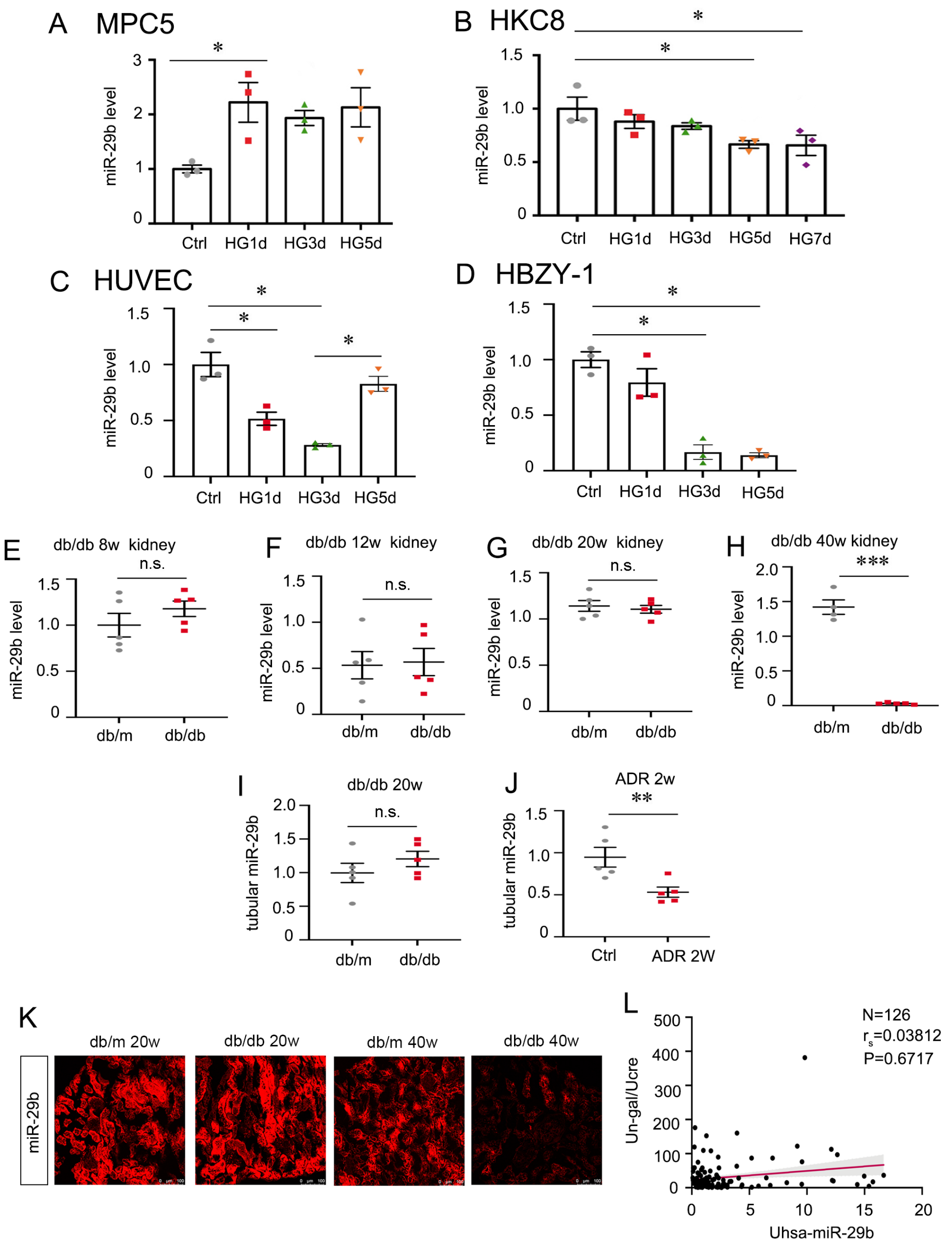

Figure S2

Supplementary Figure S2. MiR-29b expression in different conditions

(A) Graphic presentation shows the expression changes of the miR-29b with high-glucose treatment in MPC5. *$P < 0.05$ versus control group (n=3). (B) Graphic presentation shows the expression changes of the miR-29b with high-glucose treatment in HKC8. *$P < 0.05$ versus control group (n=3). (C) Graphic presentation shows the expression changes of the miR-29b

with high-glucose treatment in HUVEC. *P < 0.05 versus control group (n=3). (B) Graphic presentation shows the expression changes of the miR-29b with high-glucose treatment in HBZY-1. *P < 0.05 versus control group (n=3). (E-H) Graphic presentation shows the expression changes of the miR-29b in kidney tissues in different ages of db/db mice. ***P < 0.001 versus db/m group (n=5). (I-J) Graphic presentation shows the expression changes of the miR-29b in primary tubules in db/db of 20 weeks, ADR of 2 weeks. **P < 0.01 versus control group (n=5). (K) In situ hybridization shows the expression of miR-29b in db/db mice. (L) Linear regression shows no correlation between urinary miR-29b and N-gal level in urine (n = 126, DKD I-II stage).

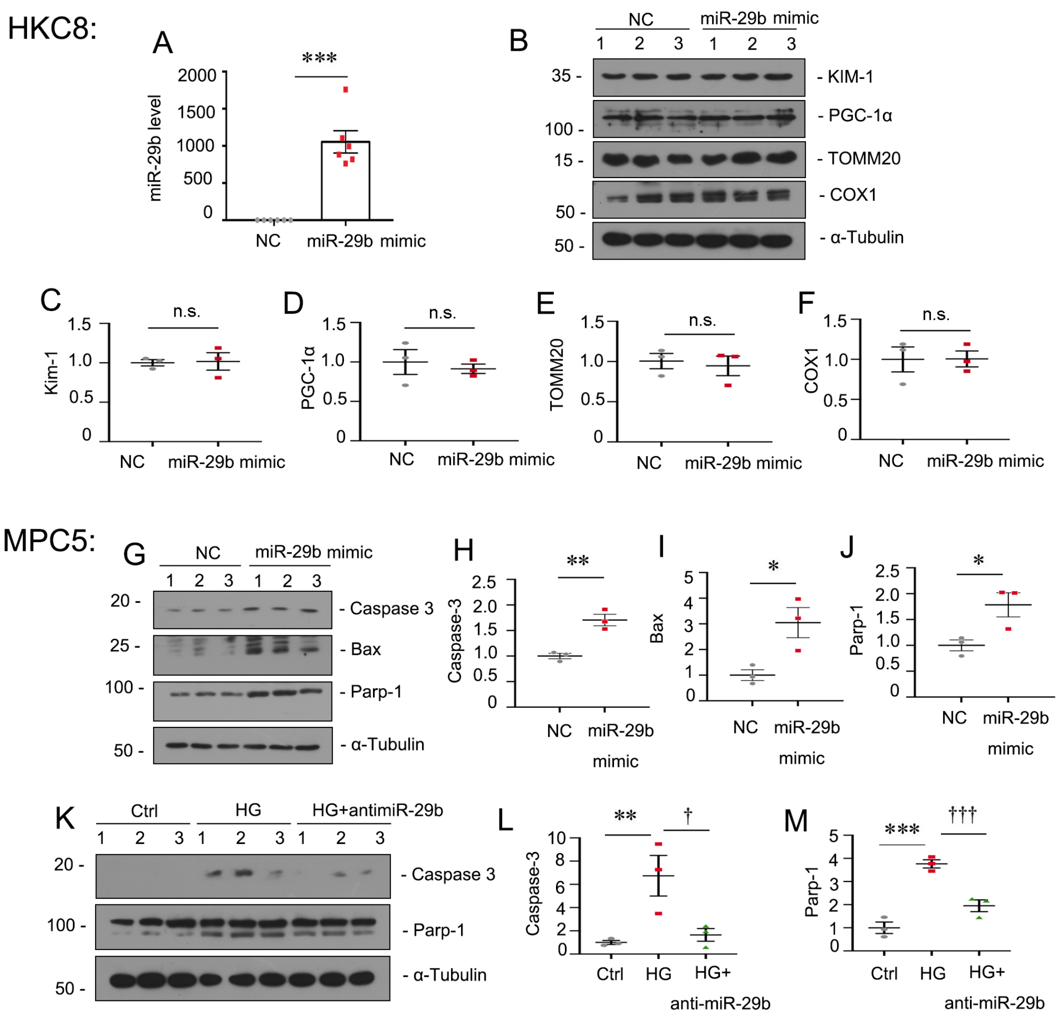

Figure S3

Supplementary Figure S3. MiR-29b induces cell injury in podocytes but not in tubular cells

(A) Graphic presentation shows the expression of miR-29b in HKC8 transfected with miR-29b mimic. ****P* < 0.001 versus control group (n=3). (B-F) Representative western blot and quantitative data showing expression of KIM-1, PGC-1α, TOMM20 and COX1 in two groups. Numbers (1–3) indicate each individual culture in each given group. (G-J) MPC5 were transfected with miR-29b mimics or negative control (NC) for 24h. Representative western blot and quantitative data showing expression of Caspase 3, Bax and Parp-1 in two groups. Numbers (1–3) indicate each individual culture in each given group. **P* < 0.05, ***P* < 0.01 versus control

group (n=3). (K-M) MPC5 were transfected with anti-miR-29b and high glucose treatment for 48h. Representative western blot and quantitative data showing expression of Caspase 3 and Parp-1 in three groups. Numbers (1–3) indicate each individual culture in each given group. ***P* < 0.01, ****P* < 0.001 versus control group (n=3); †*P* < 0.05, †††*P* < 0.001 versus high glucose group (n=3).

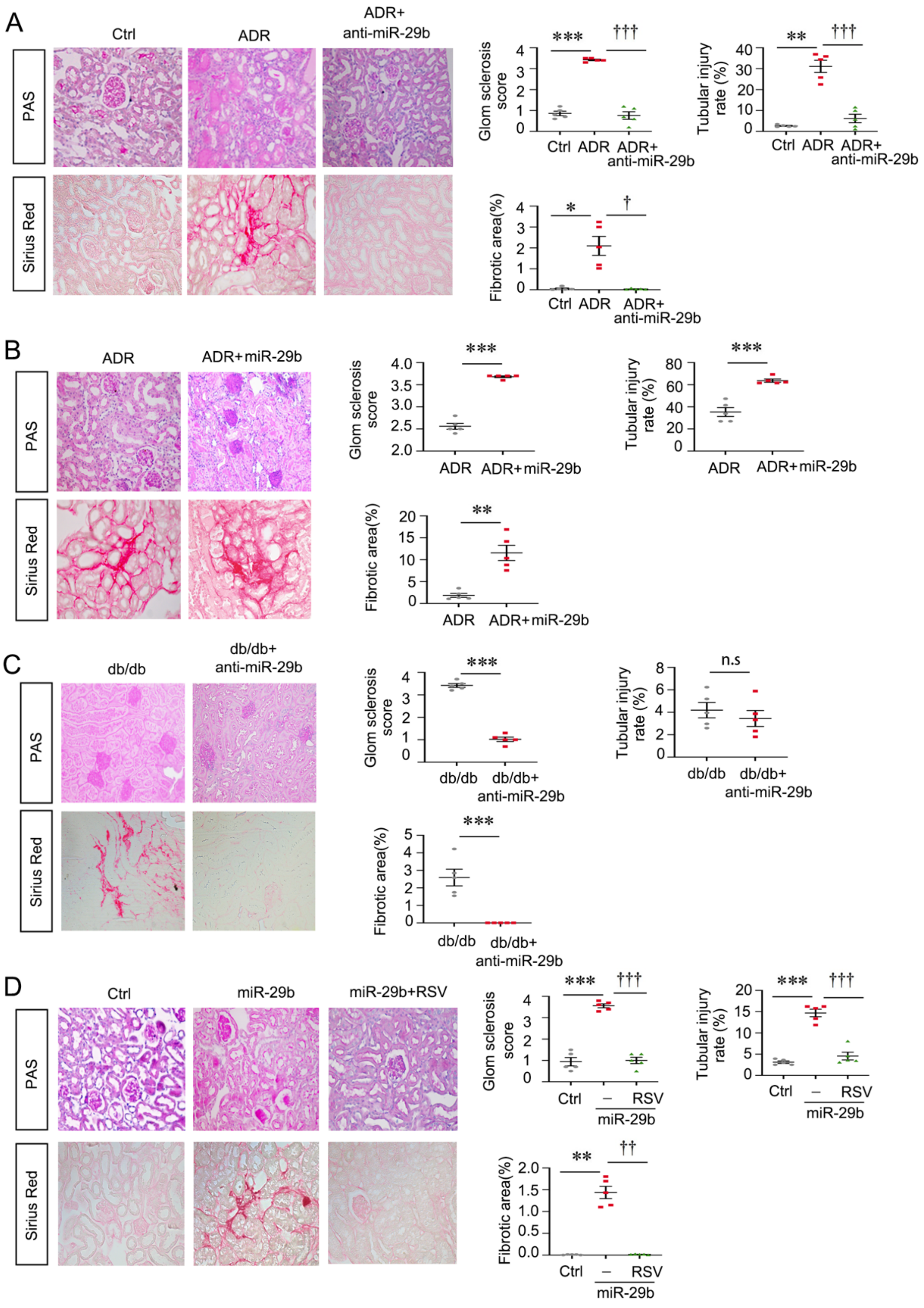

Figure S4

Supplementary Figure S4. PAS staining and Sirius Red staining

(A-D) Representative images of PAS staining and Sirius Red staining and quantitative data in different animal models.

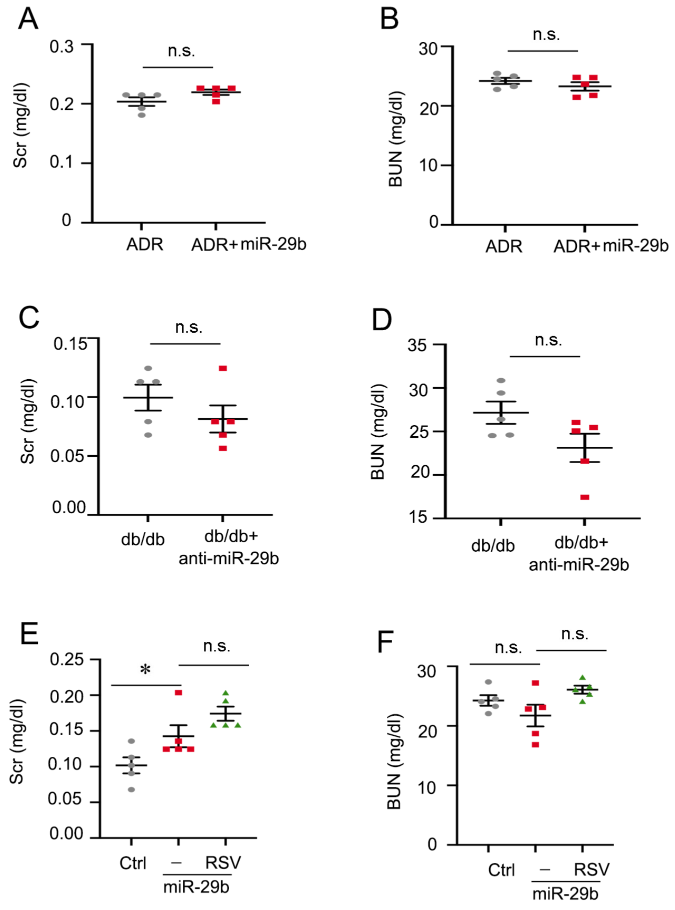

Figure S5

Supplementary Figure S5. Renal function

(A-F) Graphic presentation shows serum creatinine levels (Scr) and blood urea nitrogen (BUN) in different groups as indicated.
